# Differential expression analysis at the individual level reveals a lncRNA prognostic signature for lung adenocarcinoma

**DOI:** 10.1186/s12943-017-0666-z

**Published:** 2017-06-06

**Authors:** Fuduan Peng, Ruiping Wang, Yuanyuan Zhang, Zhangxiang Zhao, Wenbin Zhou, Zhiqiang Chang, Haihai Liang, Wenyuan Zhao, Lishuang Qi, Zheng Guo, Yunyan Gu

**Affiliations:** 1 Department of Systems Biology, College of Bioinformatics Science and Technology, Harbin Medical University, Harbin, 150086 China; 2 Training Center for Students Innovation and Entrepreneurship Education, Harbin Medical University, Harbin, 150086 China; 3 Department of bioinformatics, Key Laboratory of Ministry of Education for Gastrointestinal Cancer, Fujian Medical University, Fuzhou, 350001 China; 4 Fujian Key Laboratory of Tumor Microbiology, Fujian Medical University, Fuzhou, 350001 China; 5 Department of Pharmacology, Harbin Medical University, Harbin, 150086 China

**Keywords:** lncRNAs, differentially expressed lncRNA, Lung adenocarcinoma, Prognostic signature, Individual level

## Abstract

**Background:**

Deregulations of long non-coding RNAs (lncRNAs) have been implicated in cancer initiation and progression. Current methods can only capture differential expression of lncRNAs at the population level and ignore the heterogeneous expression of lncRNAs in individual patients.

**Methods:**

We propose a method (*LncRIndiv*) to identify differentially expressed (DE) lncRNAs in individual cancer patients by exploiting the disrupted ordering of expression levels of lncRNAs in each disease sample in comparison with stable normal ordering. *LncRIndiv* was applied to lncRNA expression profiles of lung adenocarcinoma (LUAD). Based on the expression profile of LUAD individual-level DE lncRNAs, we used a forward selection procedure to identify prognostic signature for stage I-II LUAD patients without adjuvant therapy.

**Results:**

In both simulated data and real pair-wise cancer and normal sample data, *LncRIndiv* method showed good performance. Based on the individual-level DE lncRNAs, we developed a robust prognostic signature consisting of two lncRNA (*C1orf132* and *TMPO-AS1*) for stage I-II LUAD patients without adjuvant therapy (*P* = 3.06 × 10^−6^, log-rank test), which was confirmed in two independent datasets of GSE50081 (*P* = 1.82 × 10^−2^, log-rank test) and GSE31210 (*P* = 7.43 × 10^−4^, log-rank test) after adjusting other clinical factors such as smoking status and stages. Pathway analysis showed that *TMPO-AS1* and *C1orf132* could affect the prognosis of LUAD patients through regulating cell cycle and cell adhesion.

**Conclusions:**

*LncRIndiv* can successfully detect DE lncRNAs in individuals and be applied to identify prognostic signature for LUAD patients.

**Electronic supplementary material:**

The online version of this article (doi:10.1186/s12943-017-0666-z) contains supplementary material, which is available to authorized users.

## Background

Long non-coding RNAs (lncRNAs) are non-coding RNAs ranging in length from 200 nucleotides to ~100 kilobases [1]. LncRNAs are implicated in a variety of biological processes and deregulation of lncRNAs may act as biomarkers and therapeutic targets for cancer [2]. Many studies identify the cancer-related lncRNAs using differential expression analysis methods, such as T-test, EdgeR [3] and DESeq [4], which are designed to detect the population-level differentially expressed (DE) lncRNAs. Although some methods, such as Maximum Ordered Subset T-statistic (MOST) [5], Cancer Outlier Profile Analysis (COPA) [6], Outlier Sums (OS) [7] and Outlier Robust T-statistic (ORT) [8], have already been proposed to detect differentially expressed genes (DEGs) in sub-groups of cancer samples, considering the high heterogeneity of lncRNA expression among patients, none have been used in detecting DE lncRNAs in individual patients. Recently, our research group has successfully developed new methods to detect patient-specific differential expression information [9, 10]. We have revealed that the relative expression rankings of genes (miRNAs) tend to be highly stable in specific normal human tissues but widely disturbed in the corresponding cancer tissues, and the reversal relationship of rank between genes (miRNAs) expression level can be used to identify DE genes (miRNAs) in individual patient. The advantage of the present relative ordering-based method is that it is insensitive to batch effects and data normalization and thus can directly utilize data from different datasets [9–11]. Thus, by evaluating the lncRNA expression profiles in this study, we proposed a new method (*LncRIndiv*) to detect DE lncRNAs in individual patients, which has been improved based on our original methodology that were developed to detect DE miRNAs in individuals [9].

Considerable efforts have been devoted to identify lncRNA prognostic signature for cancers using absolute expression profiles and risk score based methods [2, 12, 13]. However, due to experimental batch effects and platform differences, the score-based signatures tend to produce spurious risk classification in independent samples measured by different laboratories and are infeasible in clinical application [11]. Fortunately, we found prognostic signatures derived using the relative genes (miRNAs) expression rankings within samples, rather than the absolute expression values, are robust in independent datasets from different laboratories and platforms [9, 10]. For example, our previous work found that the expression rank change of hsa-miR-29c with hsa-miR-30b can be used as biomarker of poor overall survival for breast cancer patients [9]. Thus, the individual-level differential expression of lncRNA derived by the *LncRIndiv* method could be applied to detect the prognostic signature for cancer.

Lung adenocarcinoma (LUAD) is one of the important sub-types of lung cancer with high morbidity and mortality [14]. In this study, by a case study of LUAD, we demonstrated that *LncRIndiv* could reach good performance for individual-level analysis of deregulated lncRNAs in independent paired normal-cancer samples. And, a significant proportion of up- or down-regulated DE lncRNAs showed concordance of amplified or deleted copy number alterations, providing evidence of the high reliability of the *LncRIndiv* method. Based on the lncRNAs individual-level differentially expression analysis, we successfully developed a new prognostic signature (*C1orf132* and *TMPO-AS1*) for stage I and II LUAD patients without adjuvant therapy. This new signature does not rely on pre-setting thresholds for prognostic prediction and performed well in independent datasets.

## Methods

### Data and pre-processing

The microarray platform used in this work was Affymetrix Human Genome U133 Plus 2.0 Array (HG-U133 Plus 2.0), including the information of probes, Ensembl IDs and (or) RefSeq IDs. The information for each lncRNA, such as Ensembl ID, Ensembl transcript ID and symbol, was downloaded from the GENCODE (release 19). Meanwhile, the corresponding relationship between Ensembl transcript ID and the RefSeq ID for lncRNAs were downloaded from the HGNC database (version corresponding to GENCODE release 19). By matching those datasets, we got the symbols and RNA types for each probe. Finally, we retained the long non-coding genes and filtered them by removing discordant probes information, pseudogenes, rRNAs, tRNAs, snRNAs, snoRNAs and other short non-coding RNAs [13]. The information about microarray probes, the Ensembl IDs/Reference sequence IDs and symbols of each lncRNA have been recorded in the Additional file 1: Table S1.

Microarray datasets of LUAD (.CEL files) generated based on the HG-U133 Plus 2.0 were downloaded from Gene Expression Omnibus. The raw data for each dataset was processed using the RMA algorithm for background adjustment without normalization [11]. Then, each probe-set ID was mapped to the lncRNA annotation file. If multiple probe-sets were mapped to the same lncRNA, the expression value of the lncRNA was summarized as the mean of the values of multiple probe-sets. A set of normal and cancer samples were pooled together for selecting the significantly reversed lncRNA pairs (Additional file 2: Table S2). The GSE27262 dataset containing 25 paired cancer-normal samples (Additional file 2: Table S3) were used to evaluate the performance of *LncRindiv*. Besides, 136 stage I or II LUAD patients without adjuvant therapy with complete overall survival information were used as training dataset to derive the lncRNA prognostic signature (Additional file 2: Table S4). The 128 and 204 stage I and II LUAD samples without adjuvant therapy from GSE50081 and GSE31210 were used as validation datasets (Additional file 2: Table S4). The Atlas of Noncoding RNAs in Cancer (TANRIC) database provided the sequencing expression profiles of lncRNAs in large cohorts of 20 cancer types [15]. We acquired two independent lncRNA sequencing expression profiles of LUAD patients from the TANRIC database, including TANRIC-KOREN dataset and TANRIC-TCGA dataset. The TANRIC-KOREN dataset with 77 cancer samples and 87 control samples was used to select the significantly reversed lncRNA pairs (Additional file 2: Table S2). The lncRNAs with non-zero expression in at least 90% samples were retained for detecting stable lncRNA pair. Fifty-seven paired cancer-normal lncRNA expression profiles from TANRIC-TCGA dataset were used to evaluate the performance of *LncRindi*v (Additional file 2: Table S3) and 388 LUAD lncRNA expression profiles from TANRIC-TCGA dataset were used for the copy number alteration and expression consistence analysis. All tissue specimens were obtained before the patients receiving therapy.

The Affymetrix Genome-Wide Human SNP array 6.0 data of 429 LUAD samples was downloaded from The Cancer Genome Atlas (TCGA) database (https://cancergenome.nih.gov/), and was processed using the GISTIC 2.0 algorithm [16]. We used the default cutoff of 0.25 (25% False Discovery Rate, FDR) to select significant regions. Also, cutoffs of 0.1 and 0.05 were considered in this study. Benjamini-Hochberg multiple testing correction was used to estimate the *FDR* [17]. We used the GENCODE (release 19) annotation to investigate patterns of lncRNA copy number alterations. As Mermel *et al.* did [16], we used the cutoffs of log2 ratio > 0.1 for detecting amplifications and log2 ratio < −0.1 for detecting deletions to assign a discrete copy number alteration status for each lncRNA in each cancer sample. Level 3 mRNA expression profile detected by IlluminaHiSeq platforms were also obtained from the TCGA data portal (https://cancergenome.nih.gov/).

### Definition of stable and reversal lncRNA pairs

**Fig. 1:**
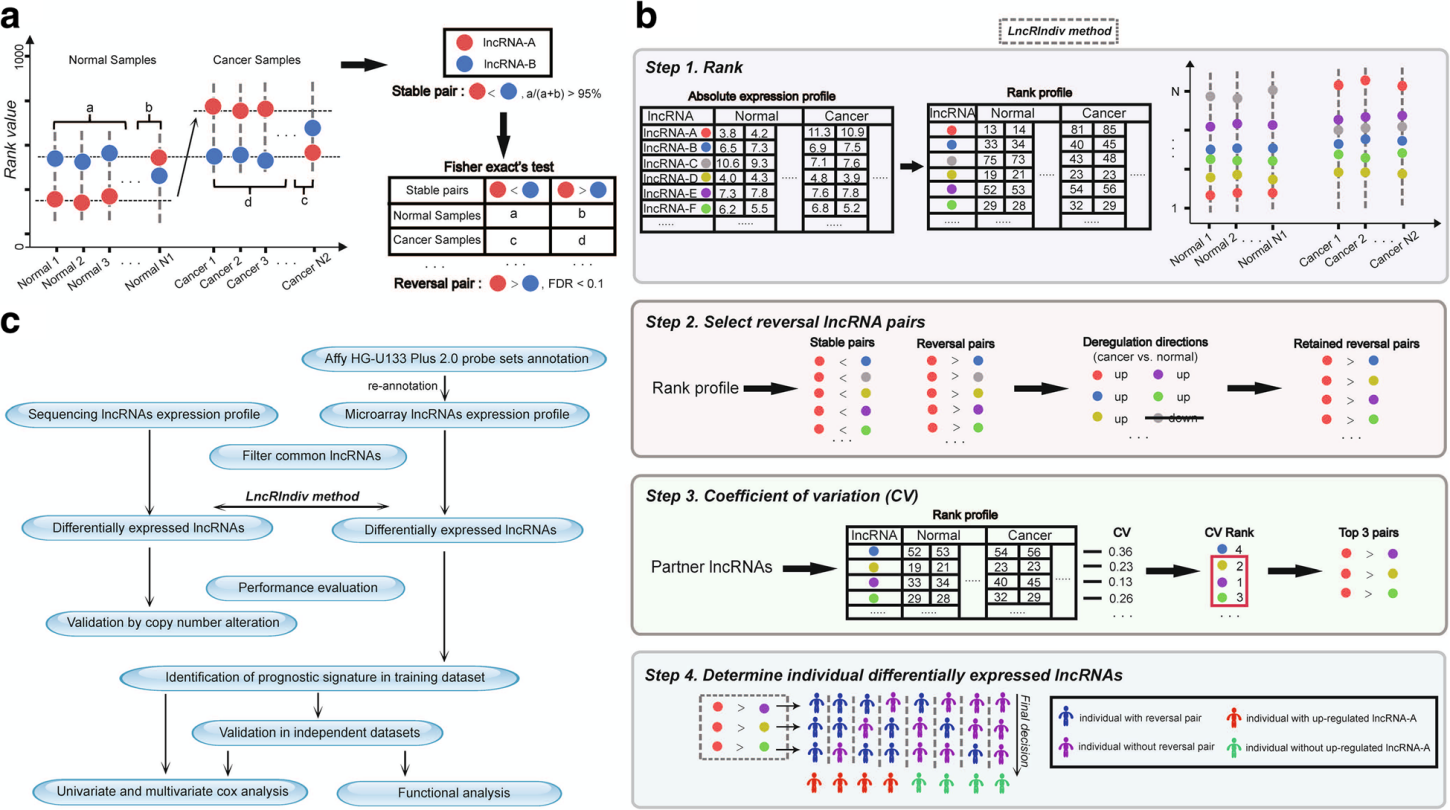
Illustration of *LncRIndiv* method and work-flow of this study. **a** Definition of stable and reversal lncRNA pairs. The red and blue circles represent *lncRNA-A* and *lncRNA-B*, respectively. The *lncRNA-A* and *lncRNA-B* are ranked according to the expression values, where the smallest expression value corresponds to the minimum rank and the greatest expression value corresponds to the maximum rank. Letters of *a* and *c* represent the number of samples with the expression level of *lncRNA-A* < *lncRNA-B* in normal and cancer samples, while *b* and *d* represent the number of samples with the expression level of *lncRNA-A* > *lncRNA-B* in normal and cancer samples, respectively. If *lncRNA-A* < *lncRNA-B* appears in more than 95% (a/(a + b) > 95%) of normal samples, *lncRNA-A* < *lncRNA-B* is selected as a stable pair. The Fisher’s exact test is used to test whether the rank of *lncRNA-A* < *lncRNA-B* is significantly reversed as *lncRNA-A* > *lncRNA-B* in cancer samples. The *P* values are corrected by Benjamini-Hochberg multiple tests and *lncRNA-A* > *lncRNA-B* with FDR < 0.1 is defined as a reversal pair. **b** The schematic diagram of *LncRIndiv* method. Take *lncRNA-A* as an example to describe the *LncRIndiv* method. Circles with different color represent different lncRNAs. The blue and purple human shapes represent the samples with or without reversal lncRNA pairs in each line, respectively. The red and green human shapes represent the samples are determined as with and without differential expression of *lncRNA-A* by the *LncRIndiv* method. See the detailed explanation of *LncRIndiv* in Method section. **c** The work-flow of this study

Each lncRNA’s expression value was converted to its rank within each sample (the smallest expression value corresponding to the minimum rank, and the greatest expression value corresponding to the maximum rank). Pairwise comparisons were performed for all lncRNAs to identify lncRNA pairs with stable order in normal samples. Stable lncRNA pairs were defined as patterns of rank, such as *lncRNA-A* < *lncRNA-B*, appearing in more than 95% of normal samples (*P* = 6.26 × 10^−23^, binomial test, Fig. 1a). Reversal lncRNA pairs were defined as lncRNA pairs that displayed a significant reversal order in cancer samples compared with their stable order in normal samples (*lncRNA-A* < *lncRNA-B* → *lncRNA-A* > *lncRNA-B*) using Fisher’s exact test at *FDR* < 0.1.

### *LncRIndiv* method

Step 1: The absolute expression profile of lncRNAs is transformed into rank profile.
Step 2: Take *lncRNA-A* as an example. According to the rules in Fig. 1a, there are five reversal pairs with *lncRNA-A* in cancer samples, including partner lncRNAs *lncRNA-B*, *lncRNA-C*, *lncRNA-D*, *lncRNA-E*, and *lncRNA-F*. Only the partner lncRNAs that have the same dysregulation directions as *lncRNA-A* in the *lncRNA-A* reversal pairs are retained. Here, the dysregulation directions indicated the expression of *lncRNA-A* is up-regulated in the cancer group comparing with the normal group. In Fig. 1b, the *lncRNA-C* is removed because of the down-regulation trend.
Step 3: Then, we calculate the coefficient of variation (CV) of rank across cancer and normal samples for each partner lncRNA of *lncRNA-A* in the *lncRNA-A* reversal pairs (Fig. 1b). We hypothesize that if the rank of partner lncRNA is approximately constant across the cancer and normal samples, the reversal relationship of *lncRNA-A* and partner lncRNA may occur because of the rank change of *lncRNA-A*, which could be used as evidence to determine whether *lncRNA-A* is differentially expressed in individual cancer samples. Then, the partner lncRNAs of *lncRNA-A* are ranked by the CV in increasing order. If there are more than 3 reversal pairs for *lncRNA-A*, the top 3 reversal pairs are retained; otherwise, all are included for the following analysis. In Fig. 1b, the *lncRNA-D*, *lncRNA-E* and *lncRNA-F* are the top three lncRNAs with the smallest CV and are included in following analysis.
Step 4: In this example, the top 3 reversal lncRNA pairs (*lncRNA-A* > *lncRNA-D*, *lncRNA-A* > *lncRNA-E* and *lncRNA-A* > *lncRNA-F*) are used to determine whether *lncRNA-A* is differentially expressed in an individual patient. If more than half of the reversal lncRNA pairs are detected in a patient, we conclude that *lncRNA-A* is differentially expressed in the patient (red human shape in Fig. 1b).

### Evaluating the performance of *LncRIndiv*

First, a simulation was performed to evaluate the performance of *LncRIndiv* method. To keep the intrinsic structure of real lncRNA data, the simulations were conducted based on the real dataset (see Results section for detailed description of simulation experiments). The simulation experiment enables us to know both the DE lncRNAs and non-DE lncRNAs and facilitates the calculation of sensitivity, specificity and F-score. Here, the sensitivity is defined as the ratio of correctly identified DE lncRNAs to all DE lncRNAs and the specificity is defined as the ratio of correctly identified non-DE lncRNAs to all non-DE lncRNAs. The F-score, a harmonic mean of sensitivity and specificity, was calculated as follows:

$$ F- score=\frac{2\left( sensitivity\times specificity\right)}{sensitivity+ specificity} $$

Moreover, the real pair-wise cancer-normal samples were used to evaluate the consistence of dysregulation directions of DE lncRNAs between those identified by *LncRIndiv* method and the actual dysregulation directions observed in the paired samples. For a pair-wise cancer and normal tissues, if the rank of a lncRNA in cancer sample was larger than that of matched normal sample, the dysregulation direction of the lncRNA was up-regulated (and vice versa), which was taken as the benchmark. The consistency score was calculated as the ratio of the observed consistent DE lncRNAs to all DE lncRNAs identified in each sample.

### Developing the prognostic lncRNA signature

First, for each DE lncRNA, stage I and II LUAD patients without adjuvant therapy were separated into with and without DE lncRNA groups. Then, we selected prognosis-related lncRNAs that were significantly associated with patient overall survival using the log-rank test [18] and univariate Cox proportional-hazards regression model (*P* < 0.05) [19]. Harrell’s concordance index (C-index) was used to quantify the predictive accuracy of the prognosis-related lncRNA. A C-index value of 0.5 indicates no predictive ability, whereas a value of 1 represents perfect predictive ability [20]. We performed a forward selection process to search a set of lncRNAs that achieved the largest C-index value based on following procedures. Step 1: rank the prognosis-related lncRNAs in a decreasing C-index value order. Step 2: choose the lncRNA with the maximal C-index as a seed of the candidate prognosis-related signature. Step 3: add a prognosis-related lncRNA to the candidate signature once at a time based on the decreasing C-index value to obtain the new candidate prognosis-related signature. Step 4: evaluate the C-index value of the new signature and keep the new added lncRNA if the C-index is increased. Step 5: repeat step 3 and 4 until the final C-index value is not increased. Finally, a set of lncRNAs with the largest C-index is chosen as the prognostic signature for stage I and II LUAD patients without adjuvant therapy. Survival curves were plotted using the Kaplan-Meier method [21].

### Functional analysis of lncRNA

T-test was used to detect the DEGs between the high- and low-risk patients at the *FDR* < 0.05, which were defined as the lncRNA-DEGs. Then, we used GO-function method to extract the biological process from Gene Ontology (GO) database that were significantly enriched with lncRNA-DEGs (*FDR* < 0.05) [22]. To investigate the regulation relationship between lncRNAs and genes, we detected the significantly co-expressed lncRNAs and DEGs in the high-risk patient group (*P* < 0.05, Pearson Correlation Test). Then, we performed the KEGG (Kyoto Encyclopedia of Genes and Genomes, Release 58.0) pathway enrichment analysis for the lncRNA correlated DEGs to study the regulation function for the lncRNA.

## Results

### Identification of reversal lncRNA pairs from microarray and sequencing expression profiles

For each LUAD sample, lncRNA expression values were converted to rank values with increasing order. A stable lncRNA pair was defined as that the rank relationships between the expression levels of two lncRNAs were presented in more than 95% of normal samples (*P* = 6.26 × 10^−23^, binomial test, Fig. 1a). There were 237459 and 213235 stable lncRNA pairs derived from the microarray dataset combined from five datasets (GSE18842, GSE37768, GSE31210, GSE19188, GSE19804) and the sequencing dataset (TANRIC-KOREN) (Additional file 2: Table S2), respectively. And, 128540 lncRNA pairs were overlapped between the two lists of stable lncRNA pairs (*P* < 1.0 × 10^−15^, hypergeometric test), indicating that stable lncRNA pairs are highly reproducible between different platforms. Compared with normal samples, 75648 and 38611 lncRNA pairs were significantly reversed in LUAD cancer samples from microarray and sequencing data, respectively. The consistent ratio of the reversal lncRNA pairs between the microarray and sequencing was 98.51% (*P* < 1.0 × 10^−15^, hypergeometric test). Thus, to evaluate the performance of *LncRIndiv* between different platforms, we performed all analysis on the common 1310 lncRNAs between microarray and sequencing datasets.

A reversal lncRNA pair was defined as a lncRNA pair that displayed a significant reversal order in cancer samples compared with its stable order in normal samples (Fisher’s exact test, *FDR* < 0.1). For a lncRNA, considering all the partner lncRNAs that have reversal relationship with the specific lncRNA, we selected the top 3 partner lncRNAs with the smallest coefficient of variation to perform the following individual analysis. Totally, 1257 and 1123 lncRNAs could be detected with differential expression status in the microarray (Additional file 3: Table S5) and sequencing datasets (Additional file 4: Table S6) for LUAD samples, respectively. We used the heatmap to visualize the pattern of expression rank of each lncRNA pair and the significance of reversal lncRNA pair based on the top 3 pair-wise reversal pairs identified from the microarray dataset and sequencing dataset (Additional file 5: Figure S1).

### Performance evaluation in simulation dataset and independent datasets

In order to retain the intrinsic structure of the data, 50 up- and 50 down-regulated lncRNAs were randomly generated from the identified up- and down-regulated lncRNAs, separately. The 210 normal samples in the microarray training dataset were used to simulate for disease samples. First, if a lncRNA was set as differentially expressed in a sample, the pair-wise simulated diseased sample was simulated by setting the different magnitudes of differential expression (log2FC = ±1.0, ±1.5, ±2.0, FC means fold change) comparing to the expression in the normal sample. Then, an average of 10 samples generated by random in which DE lncRNA was set to be differentially expressed, which was the same as the real dataset. Finally, *LncRIndiv* method showed good performance with sensitivity, specificity and F-score more than 96%, respectively (Table 1). As Wang *et al.* did [10], to determine the effect of sample size, the performance of the method was studied in the small dataset of 60 disease samples and 60 normal samples, which were extracted from the training datasets by random. As expected, similar results were observed for each scenario (Table 1). The consistence analysis also showed a high consistency score more than 93% under the criteria of top 3, 5 and 7 reversal pairs in both microarray and RNA-Seq pair-wise dataset (Additional file 6: Table S7).

**Table 1:** Sensitivity, specificity, and F-score for *LncRIndiv* method in simulated data

|Log2FC^a^|		210 vs 210	60 vs 60
1.0	F-score	1.0000	0.9569
	sensitivity	1.0000	0.9182
	specificity	1.0000	0.9990
1.5	F-score	0.9842	0.9697
	sensitivity	0.9694	0.9430
	specificity	0.9995	0.9980
2.0	F-score	0.9916	0.9873
	sensitivity	0.9839	0.9755
	specificity	0.9995	0.9994

^a^FC denotes fold change

*RankComp* was another method to detect DE genes in individual samples [10]. Here, we also compared *LncRIndiv* with *RankComp* in simulation data under the same condition. The detailed results of simulation experiments and parameter settings were presented in Additional file 6: Table S8. The results showed that F-score, sensitivity and specificity derived by *LncRIndiv* were higher than those from *RankComp* method. Moreover, the *RankComp* reached a lower consistency score at about 81% level compared with those got by *LncRIndiv* method. Also, we showed the DE lncRNAs identified by the *LncRIndiv* and *Rankcomp* in venn diagram (Additional file 5: Figure S2). The known cancer-related lncRNAs recorded in the database of Lnc2Cancer (http://www.bio-bigdata.net/lnc2cancer) are marked with symbols in the Additional file 5: Figure S2.

### Consistence between copy number alterations and differential expression of lncRNAs in individuals

Four hundred and twenty nine LUAD samples were screened using the Affymetrix Genome-Wide Human SNP array 6.0 platform. Among the 1123 DE lncRNAs derived from sequencing dataset, 285 lncRNAs were in the regions with significant amplifications or deletions in LUAD patients. One hundred and nineteen of the 285 lncRNAs showed concordant expression changes with copy number alterations in LUAD samples, which meant that the lncRNAs with amplification (deletion) showed up-regulation (down-regulation). Then, we tested whether patients with up-regulation (down-regulation) of lncRNA were significantly overlapped with patients with copy number gain (loss) (*P* < 0.05, hypergeometric test). The results showed that 61 of the 119 lncRNAs showed significantly consistent changes between copy number alteration and deregulation of expression in individual LUAD patients (Additional file 6: Table S9), which could not be expected by chance (*P* = 8.52 × 10^−6^, hypergeometric test). When selecting lncRNAs with copy number alterations using *FDR* < 0.1 and *FDR* < 0.05, DE lncRNAs also showed consistent changes between copy number alteration and deregulation of expression in individual LUAD patients (*P* = 0.041 for the threshold of *FDR* < 0.1 and *P* = 0.085 for the threshold of *FDR* < 0.05, hypergeometric test). Thus, the significant concordance between the differential expression and copy number alteration of lncRNAs indicated the high reliability of the results derived by *LncRIndiv*.

### A prognosis-related lncRNA signature for stage I and II LUAD patients without adjuvant therapy

**Fig. 2:**
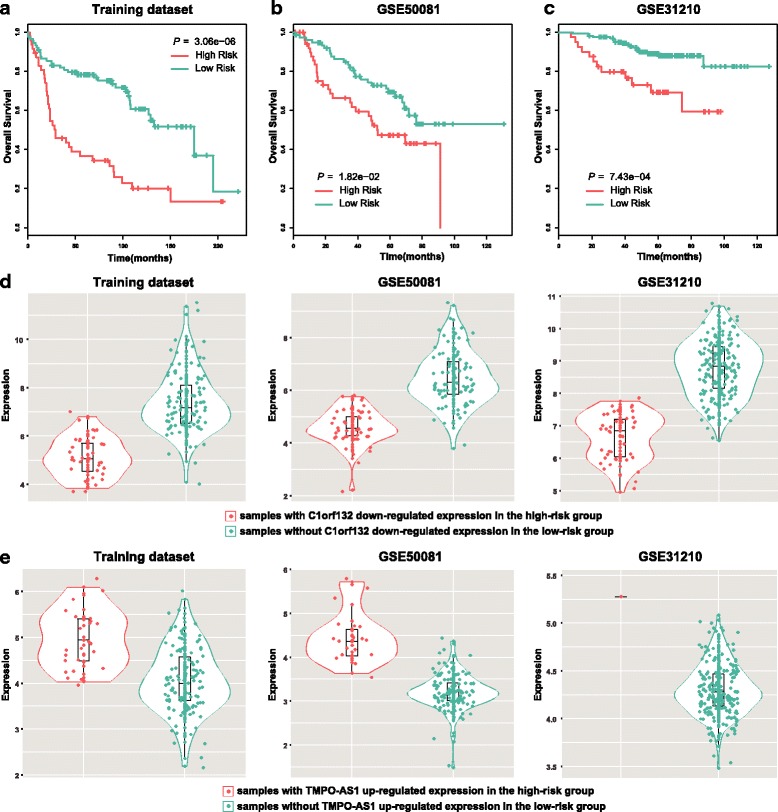
A 2-lncRNA signature for LUAD patient prognosis. Kaplan-Meier estimates the overall survival using the 2-lncRNA signature in the **a** training dataset, **b** GSE50081 and **c** GSE31210. All the *P* values of Kaplan-Meier analysis were calculated using log-rank test. **d** The lncRNA *C1orf132* was down-regulated in the high-risk patients. **e** The lncRNA *TMPO-AS1* was up-regulated in the high-risk patients

We extracted an integrated training dataset with 136 stage I or II LUAD patients without adjuvant therapy and with complete overall survival information (Additional file 2: Table S4). In total, 66 lncRNAs were significantly associated with overall survival of LUAD patients by log-rank test and univariate cox analysis (*P* < 0.05). Then, we performed a forward selection procedure to obtain a merged prognostic signature that achieved optimal prognostic performance (see Methods). As a result, a 2-lncRNA signature (*C1orf132* and *TMPO-AS1*) with a C-index of 0.641 was obtained. Patients in the high-risk group (*n* = 47) had significant shorter overall survival than those in the low-risk group (*n* = 89, *P* = 3.06 × 10^−6^, log-rank test, Fig. 2). Here, the high-risk group meant the LUAD patients with either the differential expression of *C1orf132* (down-regulation, HR = 2.27, 95% CI = (1.39, 3.71), *P* = 1.03 × 10^−3^, univariate cox analysis) or *TMPO-AS1* (up-regulation, HR = 1.89, 95% CI = (1.11, 3.22), *P* = 1.96 × 10^−2^, univariate cox analysis) and the rest LUAD patients were classified into the low-risk group. Compared with the low-risk patient group, the lncRNA *C1orf132* was down-regulated in the high-risk patients (Fig. 2d) and the lncRNA *TMPO-AS1* was up-regulated in the high-risk patients (Fig. 2e).

### Validation of the 2-lncRNA signature in independent datasets

To confirm the prognostic value of the 2-lncRNA signature, we applied the *LncRIndiv* on two independent datasets. We extracted 128 and 204 stage I or II LUAD patients without adjuvant therapy from the GSE50081 and GSE31210 datasets, respectively. For each dataset, patients were separated into high- and low-risk group based on the 2-lncRNA signature. In consistent with the findings derived from the training dataset, the 2-lncRNA signature classified 52 and 76 patients into high- and low-risk groups in GSE50081 dataset with significantly different overall survival (*P* = 1.82 × 10^−2^, log-rank test, Fig. 2b). The GSE31210 dataset was separated into 40 high- and 164 low-risk patients with significantly different overall survival (*P* = 7.43 × 10^−4^, log-rank test, Fig. 2c). The individual lncRNAs also have the prognostic value in the training and validation datasets (Additional file 5: Figure S3).

### Independence of the 2-lncRNA signature from other clinical factors

To further investigate whether the prognosis predictive ability of the 2-lncRNA signature was independent of other clinical risk factors, including age, gender, smoking status and stage, we performed the univariate and multivariate Cox regression analysis in the training dataset and two independent datasets. The 2-lncRNA signature was significantly associated with overall survival (HR = 2.59, 95% CI = (1.60, 4.18), *P* = 1.00 × 10^−4^, Table 2) in the training dataset using the univariate Cox regression test. Univariate analysis was also performed on GSE50081 (HR = 1.91, 95% CI = (1.11, 3.29), *P* = 0.020, Table 2) and GSE31210 (HR = 3.29, 95% CI = (1.58, 6.85), *P* = 1.45 × 10^−3^, Table 2) datasets. The multivariable Cox analysis showed that the 2-lncRNA signature was still significantly associated with overall survival in the training dataset (HR = 2.43, 95% CI = (1.49, 3.94), *P* = 3.45 × 10^−4^, Table 2), GSE50081 (HR = 1.82, 95% CI = (1.03, 3.22), *P* = 0.039, Table 2) and GSE31210 (HR = 2.40, 95% CI = (1.12, 5.11), *P* = 0.024, Table 2) when considering the factors of age, gender, smoking status and stage, which indicated that the 2-lncRNA signature was an independent prognostic factor for stage I and II LUAD patients without adjuvant therapy.

**Table 2:** Univariate and multivariate Cox regression analyses of the 2-lncRNA signature

Characteristics	Univariate analysis		Multivariate analysis	
	HR^a^(95% CI)	P-value	HR(95% CI)	P-value
Training dataset				
2-lncRNA signature	2.59(1.60,4.18)	1.00e–04	2.43(1.49,3.94)	3.45e–04
Age ≥ 60 vs <60 years	1.41(0.85,2.35)	0.18	1.33(0.79,2.21)	0.28
Gender female vs male	1.09(0.66,1.81)	0.74	1.10(0.66,1.84)	0.72
Stage I vs II	2.80(1.27,6.16)	0.011	2.12(0.95,4.74)	0.066
GSE50081				
2-lncRNA signature	1.91(1.11,3.29)	0.020	1.82(1.03,3.22)	0.039
Age ≥ 60 vs <60 years	1.50(0.64,3.51)	0.35	1.62(0.68,3.87)	0.27
Gender female vs male	0.74(0.43,1.28)	0.29	0.69(0.39,1.22)	0.20
Smoking vs never-smoking	1.31(0.73,2.35)	0.36	1.07(0.58,1.97)	0.83
Stage I vs II	2.54(1.45,4.44)	1.16e–03	2.49(1.40,4.41)	1.83e–03
GSE31210				
2-lncRNA signature	3.29(1.58,6.85)	1.45e–03	2.40(1.12,5.11)	0.024
Age ≥ 60 vs <60 years	1.47(0.70,3.10)	0.31	1.59(0.76,3.37)	0.22
Gender female vs male	0.59(0.29,1.22)	0.16	0.97(0.36,2.61)	0.95
Smoking vs never-smoking	1.91(0.92,3.97)	0.084	1.60(0.59,4.33)	0.35
Stage I vs II	4.30(2.09,8.83)	7.21e–05	3.42(1.62,7.26)	1.31e–03

^a^HR, hazard ratio

### Functional analysis of the lncRNA signature

Furthermore, we used T-test to detect DEGs between the high- and low-risk patients in each dataset (GSE50081 and GSE31210) at the *FDR* < 0.05, respectively. We found that 96.38% of overlapped genes between the two DEGs lists were consistent in their deregulation directions (up-regulation or down-regulation). Next, using the GO-function method [22], we further found that the DEGs detected from GSE50081 and GSE31210 were enriched in 139 and 123 biological process terms derived from the GO database, respectively (*FDR* < 0.05). Twenty-four biological process terms overlapped between the two term lists, including “DNA replication”, “cell cycle”, and “cell division” and so on, which couldn’t be expected by chance (*P* < 1.0 × 10^−15^, hypergeometric test). Results of highly overlapping DEGs and GO terms between the two datasets suggest that the 2-lncRNA is a robust prognostic signature for stage I or II LUAD patients without adjuvant therapy.

**Fig. 3:**
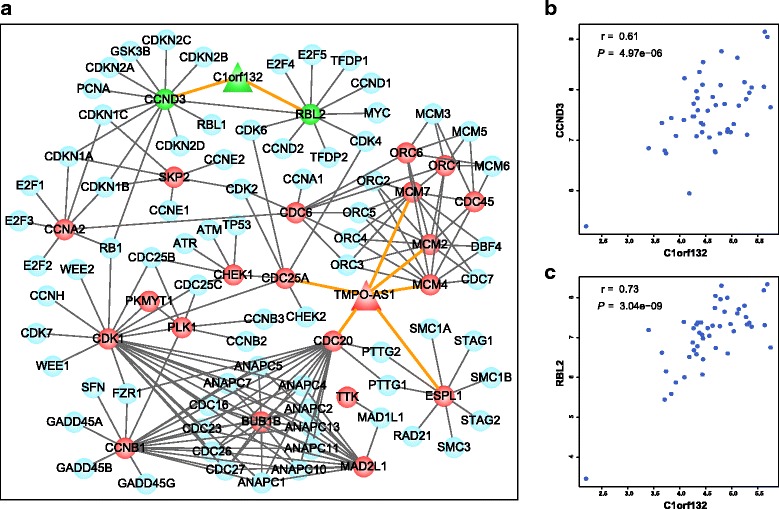
Sub-network of cell cycle pathway regulated by *C1orf132* and *TMPO-AS1*. **a** The triangles represent lncRNAs. The circles represent the cell cycle pathway genes. Nodes with red and green color represent that the genes or lncRNAs were up-regulated and down-regulated in high-risk LUAD patients compared with low-risk LUAD patients. The gray circles represent the genes that directly interact with differentially expressed genes in the cell cycle pathway, which are marked by gray lines. The orange lines represent the significantly co-expressed relationships between lncRNAs and differentially expressed genes. **b** Expression correlation between *C1orf132* and *CCND3*. **c** Expression correlation between *C1orf132* and *RBL2*. The *P* values were calculated using Pearson Correlation test in (**b**) and (**c**)

Moreover, GO enrichment results suggest that the two lncRNAs may regulate the “cell cycle”, “cell division” of cancer cells to affect the prognosis of LUAD patients. Thus, we further investigated the regulation relationship between lncRNAs and lncRNA-DEGs, which might be the potential mechanism to induce the poor prognosis of LUAD. Based on the GO-function analysis, we performed the KEGG pathway enrichment using the overlapped DEGs between the high- and low-risk groups derived from the GSE31210 and GSE50081. Under the control of *FDR* < 0.05, “Cell cycle” pathway (*P* = 2.35 × 10^−7^, hypergeometric test) and “Cell adhesion molecules (CAMs)” pathway (*P* = 9.75 × 10^−4^, hypergeometric test) were significantly enriched with the lncRNA-DEGs. Twenty-two DEGs were annotated in the cell cycle pathway (Additional file 5: Figure S4). In the GSE50081 dataset, for each lncRNA in the 2-lncRNA signature, we calculated the expression correlation between the lncRNA and 22 DEGs in cell cycle pathway in the high-risk patient group. The signal transduction relationship between genes in KEGG pathway was transformed into undirected gene interaction network. The sub-network consisting of the significantly co-expressed relationship between the signature lncRNAs and DEGs (*P* < 0.05), and interactions between DEGs and their first neighbors were presented in Fig. 3a.

Some of the DEGs whose expressions were significantly correlated with the lncRNAs have been reported with the critical roles in the carcinogenesis. For example, over-expression of gene *CDC25A*, an oncogene, was significantly correlated with poor overall survival in non-small cell lung cancer [23]. In our results, gene *CDC25A* was positively co-expressed with lncRNA *TMPO-AS1* (*P* = 3.25 × 10^−2^, Additional file 6: Table S10) and *CDC25A* was significantly up-regulated in the high-risk patients compared with the low-risk patients (*P* = 1.58 × 10^−5^, T-test, Additional file 6: Table S10). Moreover, over-expression of gene *CDC20* could predict poor prognosis in primary non-small cell lung cancer patients [24]. Our results showed that *TMPO-AS1* was also positively correlated with *CDC20* (*P* = 4.14 × 10^−2^, Additional file 6: Table S10) and *CDC20* was significantly up-regulated in the high-risk patients compared with the low-risk patients (*P* = 7.46 × 10^−6^, T-test, Additional file 6: Table S10). Some studies reported that *RBL2* [25] and *CCND3* may behave as tumor suppressors in LUAD [26]. In our study, the expression of *RBL2* and *CCND3* were significantly suppressed (*P* = 3.58 × 10^−3^ for *RBL2*, *P* = 1.33 × 10^−6^ for *CCND3*, T-test, Additional file 6: Table S10) in the high-risk LUAD patients and both of them were significantly positive co-expressed with the *C1orf132* (*P* = 3.04 × 10^−9^ for *RBL2* and *P* = 4.97 × 10^−6^ for *CCND3*, Fig. 3b and c) in the GSE50081 dataset. The significant correlation between *C1orf132* and *CCND3*, *RBL2* also happened in the GSE31210 dataset. Because *TMPO-AS1* was only detected with differential expression in one patient in the GSE31210 dataset, we did not perform correlation analysis for *TMPO-AS1* in this dataset. Based on the above results, we inferred that the two lncRNAs could affect the prognosis of LUAD by regulating the cell cycle pathway. Moreover, as the analysis process for cell cycle pathway, we also found the *C1orf132* could significantly regulate the cell adhesion molecules (*P* = 9.75 × 10^−4^, hypergeometric test, Additional file 5: Figure S5 and S6), which indicates that the lncRNA *C1orf132* may be involved in the poor prognosis of LUAD patients by promoting the invasive process of cancer cells.

Availability and Implementation: *LncRIndiv* is developed using R-3.1.2 (https://www.R-project.org) and is freely available in https://github.com/FuduanPeng/LncRIndiv (LncRIdiv_1.0.zip for Windows system and LncRIndiv_1.0.tar.gz for Linux system).

## Discussion

Aberrant expressions of lncRNAs in cancer patients have been comprehensively reported [27]. The expression levels of lncRNAs across the patients in the same cancer type are also highly heterogeneous. Current methods to detect DE lncRNAs are based on the population rather than individuals. Based on our previous study of detecting the DE miRNAs in individuals [9], we provided a new method *LncRIndiv* to detect the DE lncRNAs in individual cancer patients, which is not limited by the platform, data normalization methods and batch effects. In the method *LncRIndiv*, we used the CV of rank rather than the absolute expression levels of partner lncRNAs in our previous work [9], which could avoid the batch effect from different datasets. Notably, absolute expression values rather than the rank can actually reflect the differential expression direction of each lncRNA itself in the pair-wise cancer and normal samples. Thus, we used the expression rank of lncRNAs in pair-wise sample to evaluate the performance of *LncRIndiv* method. The *LncRIndiv* performed well in the independent pairwise LUAD datasets and the simulation data.

In our work, the *LncRIndiv* method also identified some DE lncRNAs that were well characterized by other studies (Additional file 3: Table S5, Additional file 4: Table S6, Additional file 5: Figure S1B and S2). For example, Hou *et al.* revealed that enhanced expression of long non-coding RNA *ZXF1*, known as *ACTA2-AS1* (Ensembl ID: ENSG00000180139.10) (Additional file 6: Table S11), promoted the invasion and migration of LUAD cells [28]. *LINC01207*, also named as *RP11-294O2.2* (Ensembl ID: ENSG00000248771.1) (Additional file 6: Table S11), was significantly up-regulated in advanced LUAD and the siRNA mediated knockdown of *LINC01207* in A549 cell line could inhibit the cell proliferation [29]. Some differential expression profile of lncRNAs in individuals could be partly validated by the copy number alterations of lncRNAs in individuals. As Yan *et al*. pointed that the copy number alteration is an important mechanism that leads to the aberrant expression of lncRNAs in cancer [27]. For example, the deregulation of lncRNA *BCAL8* showed positive correlation with its copy number alteration and was significantly associated with poor survival in breast cancer [27]. In our results, 51.3% lncRNAs showed significantly consistent changes between copy number alteration and deregulation of expression in individual LUAD patients. Some DE lncRNAs with consistent copy number alteration in our results have been proved to be tumor suppressor or oncogenic lncRNAs in cancer (Additional file 6: Table S9). For example, Yao *et al.* found that the down-expression of *ADAMTS9-AS2* resulted in a significant loss in the inhibition of glioma cell migration [30]. These results not only suggested that the differential expression of lncRNAs in individuals could be owing to the copy number alteration of itself, but also could be evidence to support the high reliability of individual lncRNA differentially expressed profile derived by the *LncRIndiv* method. Notably, the rest of lncRNAs without significant consistence between differential expression and copy number alterations maybe affected by mutation, methylation and so on, which warrants our future work.

Some studies use the average or median score or the expression level as cut-offs to distinguish high- and low-risk patients [13, 31–33]. However, these methods are arbitrary in setting a threshold for prognostic signature detection and are difficult to apply to clinical experiments [11, 34]. Our study reveals a robust 2-lncRNA signature for LUAD patients, which was validated in independent datasets and also by the GO enrichment analysis. In clinical translational application, for each individual LUAD patient, we only need to test whether the expression of *C1orf132* is lower than *IQCH-AS1*, *RP11-589P10.5* and *LINC00938*, or the expression of *TMPO-AS1* is higher than *PCBP1-AS1*, *TCL6* and *RP11-333E1.1*. By pathway analysis, our results suggest that the lncRNAs in the signature are involved in the poor prognosis of LUAD patients by deregulating the cell cycle and cell adhesion molecules pathways in cancer cells, which deserves our future detailed biological experiments. Notably, our results also found the stage is a factor that related with the prognosis of LUAD patients. However, as shown in Table 2, the multivariate cox analysis showed that the 2-lncRNA signature is independent of the clinical factor of stage.

In our study, we found the down-regulation of *C1orf132* was associated with the poor prognosis. The underline mechanism is still unclear. It has been proposed that lncRNAs can act as competing endogenous RNAs (ceRNAs) to influence miRNA activity and thereby regulate the target transcripts containing miRNA-binding sites [35]. We supposed that *C1orf132* may act as ceRNA with the tumor suppressors *RBL2* and *CCND3*, which have been showed with significant positive correlation with the expression of *C1orf132* in the (Fig. 3b and c). By integrating the lncRNA-miRNA interactions and miRNA-target interactions in databases of miRanda [36], miRTarBase [37], miRcode [38] and TargetScan [39], we found *C1orf132* was significantly competitively binding miRNAs with *RBL2* (*P* = 2.38 × 10^−12^, hypergeometric test) and *CCND3* (*P* = 9.82 × 10^−5^, hypergeometric test) (Additional file 6: Table S12). Some miRNAs, such as hsa-miR-93 [40], hsa-miR-372 [41], hsa-miR-424 [42], have been reported the important roles in the progression of LUAD. Thus, we inferred that the down-regulation of *C1orf132* might release the miRNAs that targeted *RBL2* and *CCND3* and further promote the tumor progression, which warrant further in-depth experimental research.

Nevertheless, our present method also has some limitations. First of all, although the consistency score are relatively high, *LncRIndiv* method may have insufficient power to detect all samples with differential expression of one lncRNA. We performed the *LncRIndiv* method on the simulated data with large number of samples with pre-set DE lncRNAs, the sensitivity decreased as the increased number of DE samples (Additional file 6: Table S13), which indicates *LncRIndiv* method may have insufficient power to detect all samples with one DE lncRNA. However, for each sample, though a certain number of DE lncRNAs may be missed, a significantly high proportion of lncRNAs show consistent expression changes with their copy number alterations, which indicates that the DE lncRNAs in individual patients captured by our method are true. Improving the power of *LncRIndiv* warrants our future detailed work. Secondly, we used the pair-wise cancer and normal samples to evaluate the performance of *LncRIndiv* method, which is lack of strict statistical justification. Thus, we further assessed the differential extent of lncRNAs identified by *LncRIndiv* method, based on the hypothesis that the higher the differential extents are, the less the random errors are. The fold changes of lncNRAs in patients with DE lncRNAs were significantly higher than those patients without the DE lncRNAs (*P* < 2.0 × 10^−16^, T-test). As examples shown in Additional file 5: Figure S7, the patients with the DE lncRNA showed bigger difference with the paired normal samples in expression values than the patients without the DE lncRNA. Thirdly, our work only analyzed the overlapped lncRNAs between microarray and sequencing datasets. Because of the number of lncRNAs re-annotated from the microarray is limited, results showed that the number of DE lncRNAs in individual patients from microarray and sequencing datasets are different. Although some lncRNAs were lost in the microarray, the results derived by the *LncRIndiv* method could reveal a new robust prognosis-related lncRNA signature for stage I or II LUAD patients without adjuvant therapy, which was validated in other independent microarray datasets. The *LncRIndiv* method could also be used in other cancer types with abundant sequencing expression profile of lncRNAs. Finally, by KEGG pathway enrichment and correlations analysis between lncRNAs and DEGs, we found that the lncRNAs (*TMPO-AS1* and *C1orf132*) could affect the prognosis of LUAD by deregulating cell cycle pathway genes. Although the results are interesting and meaningful, it is lack of biological experiments for further validation. We will continue to investigate the biological mechanisms that how the lncRNAs regulate the cell cycle genes during the carcinogenesis in our future work.

## Conclusions

We developed a rank-based method that was not limited by expression platforms or normalization techniques to detect differentially expressed lncRNAs in individual LUAD patients and reached good performance in both simulated data and real data. The up-regulation (down-regulation) of lncRNAs in individual LUAD samples, were significantly consistent with the copy number amplifications (deletions), supporting the DE lncRNAs detected in individuals by *LncRIndiv*. Based on the differential expression profiles of lncRNAs in individual LUAD patients derived by our method, we identified a new robust lncRNA prognostic signature consisting of *C1orf132* and *TMPO-AS1* for stage I and II LUAD patients without adjuvant therapy. This new signature did not rely on pre-setting thresholds for prognostic prediction and performed well in independent datasets.

## Additional files

Additional file 1: Table S1. Information of probes, Ensembl ID/RefSeq ID and symbol for each lncRNA annotated from Affymetrix Human Genome U133 Plus 2.0 Array (HG-U133 Plus 2.0). (XLSX 151 kb)

Additional file 2: **Table S2.** The LUAD datasets used for application of *LncRIndiv*. **Table S3.** The paired normal-cancer LUAD sample data used for evaluating the performance of *LncRIndiv*. **Table S4.** The datasets of stage I and II LUAD patients without adjuvant therapy. (DOC 95 kb)

Additional file 3: Table S5. Detail information of differentially expressed lncRNAs identified based on microarray data. (XLSX 275 kb)

Additional file 4: Table S6. Detail information of differentially expressed lncRNAs identified based on RNA-Seq data. (XLSX 230 kb)

Additional file 5: **Figure S1.** The hetamap of differentially expressed (DE) lncRNAs for microarray data (A) and sequencing data (B), respectively. **Figure S2.** Venn diagram to show the overlapped differentially expressed lncRNAs identified by *LncRIndiv* and *RankComp* using microarray data (A) and sequencing data (B). **Figure S3.** Kaplan-Meier estimates the overall survival in the training dataset and two independent validation datasets based on the differential expression of (A) *C1orf132* and (B) *TMPO-AS1*, respectively. **Figure S4.** Cell cycle pathway annotated with differentially expressed genes. **Figure S5.** Cell adhesion molecules pathway annotated with differentially expressed genes. **Figure S6.** Sub-network of cell adhesion molecules pathway regulated by *C1orf132*. **Figure S7.** Expression levels of lncRNAs in the pair-wise LUAD patients for (A) *LINC00341* and (B) *AC005083.1*. (DOC 3470 kb)

Additional file 6: **Table S7.** The consistency score under top 3, 5 and 7 reversal pairs in pair-wise datasets. **Table S8.** Comparison of *LncRIndiv* and *RankComp* methods using simulation data. **Table S9.** Information of lncRNAs with significant consistence between differential expression status and copy number alteration. **Table S10.** Information of genes co-expressed with *TMPO-AS1* and *C1orf132* in cell cycle pathway in GSE50081. **Table S11.** Differentially expressed lncRNAs identified by *LncRIndiv* method supported by experimental evidence. **Table S12.** Information of competing endogenous RNA and miRNA with the lncRNA *C1orf132*. **Table S13.**. Sensitivity, specificity, and F-score in simulated data under different scenarios. (DOC 257 kb)

## Abbreviations

C-index: Harrell’s concordance index
CV: Coefficient of variation
DE: Differentially expressed
DEGs: Differentially expressed genes
FDR: False Discovery Rate
FP: False positive
GO: Gene Ontology
KEGG: Kyoto Encyclopedia of Genes and Genomes
lncRNAs: Long non-coding RNAs
LUAD: Lung adenocarcinoma
TANRIC: The Atlas of Noncoding RNAs in Cancer
TCGA: The Cancer Genome Atlas
TP: True positive
