# Supplementary material for: Differential expression analysis at the individual level reveals a lncRNA prognostic signature for lung adenocarcinoma
Source: Mol Cancer. 2017 Jun 6;16:98. doi: 10.1186/s12943-017-0666-z (PMC5461634; doi:10.1186/s12943-017-0666-z)
Supplement: Supplementary file 2 — Table S2. The LUAD datasets used for application of LncRIndiv. Table S3. The paired normal-cancer LUAD sample data used for evaluating the performance of LncRIndiv. Table S4. The datasets of stage I and II LUAD patients without adjuvant therapy. (DOC 95 kb) [file 12943_2017_666_MOESM2_ESM.doc]

**Table S2.** The LUAD datasets used for application of *LncRIndiv.*

| Data type | Data source | Normal | Cancer | Reference |
| --- | --- | --- | --- | --- |
| Microarray | GSE18842 | 45 | / |  |
|  | GSE37768 | 20 | / | **-** |
|  | GSE31210 | 20 | / |  |
|  | GSE19188 | 65 | 45 |  |
|  | GSE19804 | 60 | 60 |  |
|  | GSE30219 | / | 81 |  |
|  | GSE29013 | / | 11 |  |
|  | GSE31546 | / | 13 | **-** |
|  | GSE37745 | / | 40 |  |
| Sequencing | TANRIC-KOREN | 77 | 87 |  |

**Table S3.** The paired normal-cancer LUAD sample data used for evaluating the performance of *LncRIndiv.*

| Data type | Data source | Normal | Cancer | Reference |
| --- | --- | --- | --- | --- |
| Microarray | GSE27262 | 25 | 25 |  |
| Sequencing | TANRIC-TCGA | 57 | 57 |  |

**Table S4.** The datasets of stage I and II LUAD patients without adjuvant therapy.

| Data type | Application | Data source | Cancer | Reference |
| --- | --- | --- | --- | --- |
| Microarray | Training dataset | GSE30219 | 80 |  |
|  |  | GSE29013 | 8 |  |
|  |  | GSE31546 | 13 | **-** |
|  |  | GSE37745 | 35 |  |
|  | Validation dataset | GSE31210 | 204 |  |
|  |  | GSE50081 | 128 |  |

**Reference**

1. Sanchez-Palencia A, Gomez-Morales M, Gomez-Capilla JA, Pedraza V, Boyero L, Rosell R et al. Gene expression profiling reveals novel biomarkers in nonsmall cell lung cancer. International journal of cancer. 2011;129(2):355-64.

2. Okayama H, Kohno T, Ishii Y, Shimada Y, Shiraishi K, Iwakawa R et al. Identification of genes upregulated in ALK-positive and EGFR/KRAS/ALK-negative lung adenocarcinomas. Cancer Res. 2012;72(1):100-11.

3. Hou J, Aerts J, den Hamer B, van Ijcken W, den Bakker M, Riegman P et al. Gene expression-based classification of non-small cell lung carcinomas and survival prediction. PloS one. 2010;5(4):e10312.

4. Lu TP, Tsai MH, Lee JM, Hsu CP, Chen PC, Lin CW et al. Identification of a novel biomarker, SEMA5A, for non-small cell lung carcinoma in nonsmoking women. Cancer epidemiology, biomarkers & prevention : a publication of the American Association for Cancer Research, cosponsored by the American Society of Preventive Oncology. 2010;19(10):2590-7.

5. Rousseaux S, Debernardi A, Jacquiau B, Vitte AL, Vesin A, Nagy-Mignotte H et al. Ectopic activation of germline and placental genes identifies aggressive metastasis-prone lung cancers. Science translational medicine. 2013;5(186):186ra66.

6. Xie Y, Xiao G, Coombes KR, Behrens C, Solis LM, Raso G et al. Robust gene expression signature from formalin-fixed paraffin-embedded samples predicts prognosis of non-small-cell lung cancer patients. Clinical cancer research : an official journal of the American Association for Cancer Research. 2011;17(17):5705-14.

7. Botling J, Edlund K, Lohr M, Hellwig B, Holmberg L, Lambe M et al. Biomarker discovery in non-small cell lung cancer: integrating gene expression profiling, meta-analysis, and tissue microarray validation. Clinical cancer research : an official journal of the American Association for Cancer Research. 2013;19(1):194-204.

8. Li J, Han L, Roebuck P, Diao L, Liu L, Yuan Y et al. TANRIC: An Interactive Open Platform to Explore the Function of lncRNAs in Cancer. Cancer Res. 2015;75(18):3728-37.

9. Wei TY, Juan CC, Hisa JY, Su LJ, Lee YC, Chou HY et al. Protein arginine methyltransferase 5 is a potential oncoprotein that upregulates G1 cyclins/cyclin-dependent kinases and the phosphoinositide 3-kinase/AKT signaling cascade. Cancer science. 2012;103(9):1640-50.

10. Der SD, Sykes J, Pintilie M, Zhu CQ, Strumpf D, Liu N et al. Validation of a histology-independent prognostic gene signature for early-stage, non-small-cell lung cancer including stage IA patients. Journal of thoracic oncology : official publication of the International Association for the Study of Lung Cancer. 2014;9(1):59-64.
