# Supplementary material for: Differential expression analysis at the individual level reveals a lncRNA prognostic signature for lung adenocarcinoma
Source: Mol Cancer. 2017 Jun 6;16:98. doi: 10.1186/s12943-017-0666-z (PMC5461634; doi:10.1186/s12943-017-0666-z)
Supplement: Supplementary file 5 — Figure S1. The hetamap of differentially expressed (DE) lncRNAs for microarray data (A) and sequencing data (B), respectively. Figure S2. Venn diagram to show the overlapped differentially expressed lncRNAs identified by LncRIndiv and RankComp using microarray data (A) and sequencing data (B). Figure S3. Kaplan-Meier estimates the overall survival in the training dataset and two independent validation datasets based on the differential expression of (A) C1orf132 and (B) TMPO-AS1, respectively. Figure S4. Cell cycle pathway annotated with differentially expressed genes. Figure S5. Cell adhesion molecules pathway annotated with differentially expressed genes. Figure S6. Sub-network of cell adhesion molecules pathway regulated by C1orf132. Figure S7. Expression levels of lncRNAs in the pair-wise LUAD patients for (A) LINC00341 and (B) AC005083.1. (DOC 3470 kb) [file 12943_2017_666_MOESM5_ESM.doc]

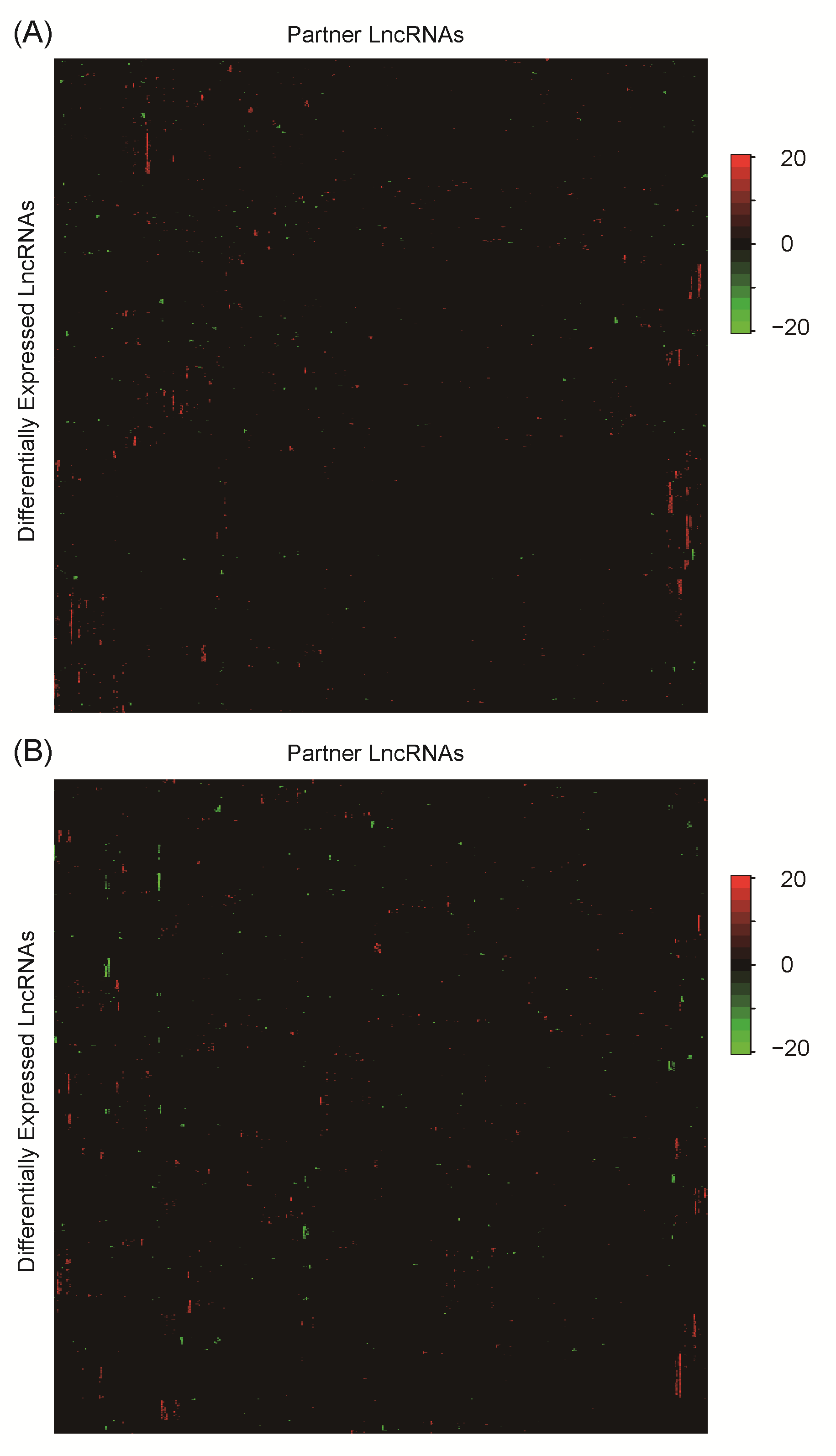


**Figure S1.** The hetamap of differentially expressed (DE) lncRNAs for microarray data (A) and sequencing data (B), respectively. Rows and columns represent DE lncRNAs and partner lncRNAs, respectively. Color corresponds to the -log10 transformed *P* value calculated by the Fisher’s exact test. Red and green color represent that the expression level of DE lncRNAs were higher and lower than partner lncRNAs in cancer patients, respectively.


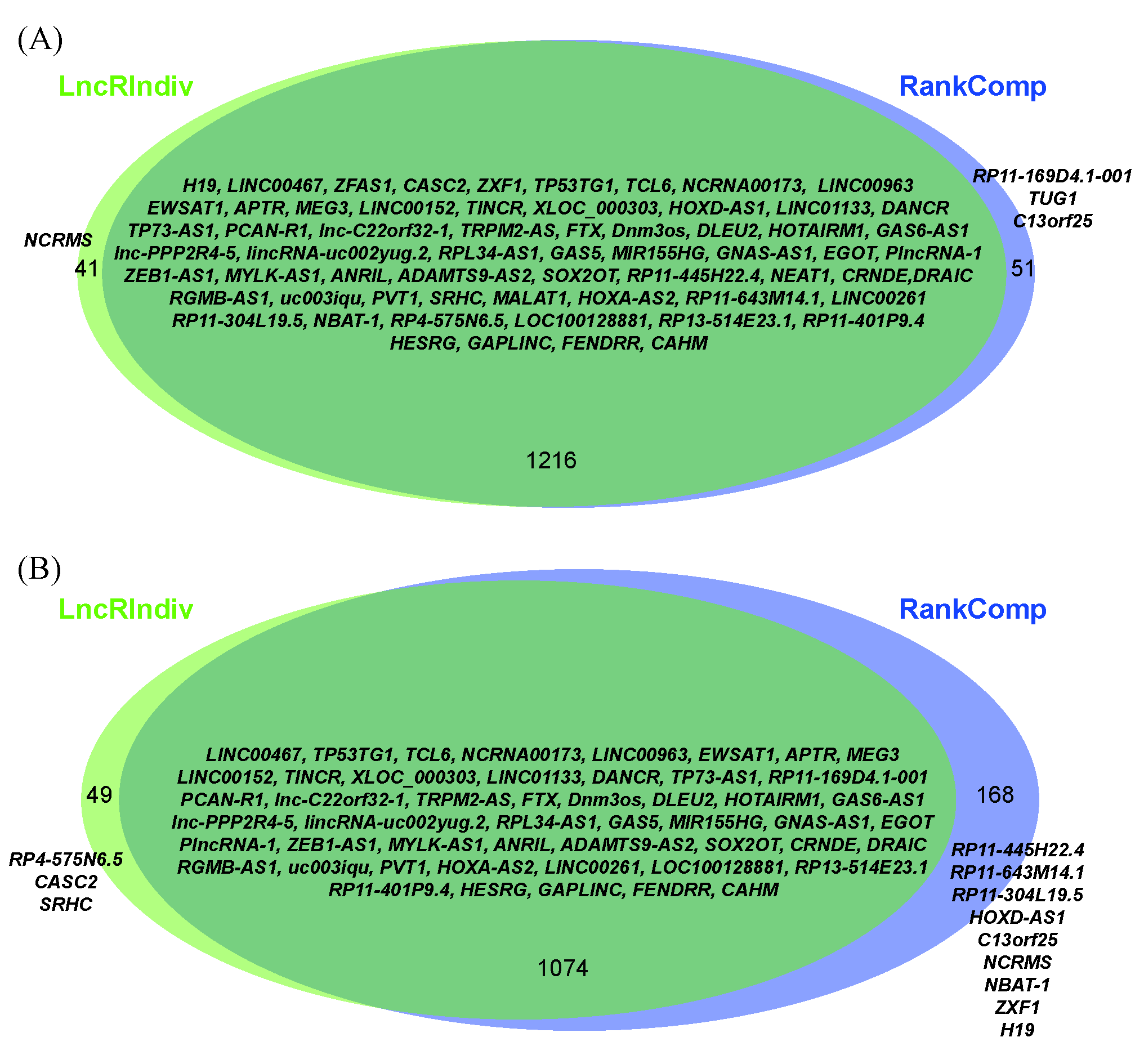


**Figure S2.** Venn diagram to show the overlapped differentially expressed lncRNAs identified by *LncRIndiv* and *RankComp* using microarray data (A) and sequencing data (B). The known cancer-related lncRNAs recorded in the database of Lnc2Cancer (http://www.bio-bigdata.net/lnc2cancer) are marked in the Venn diagram.


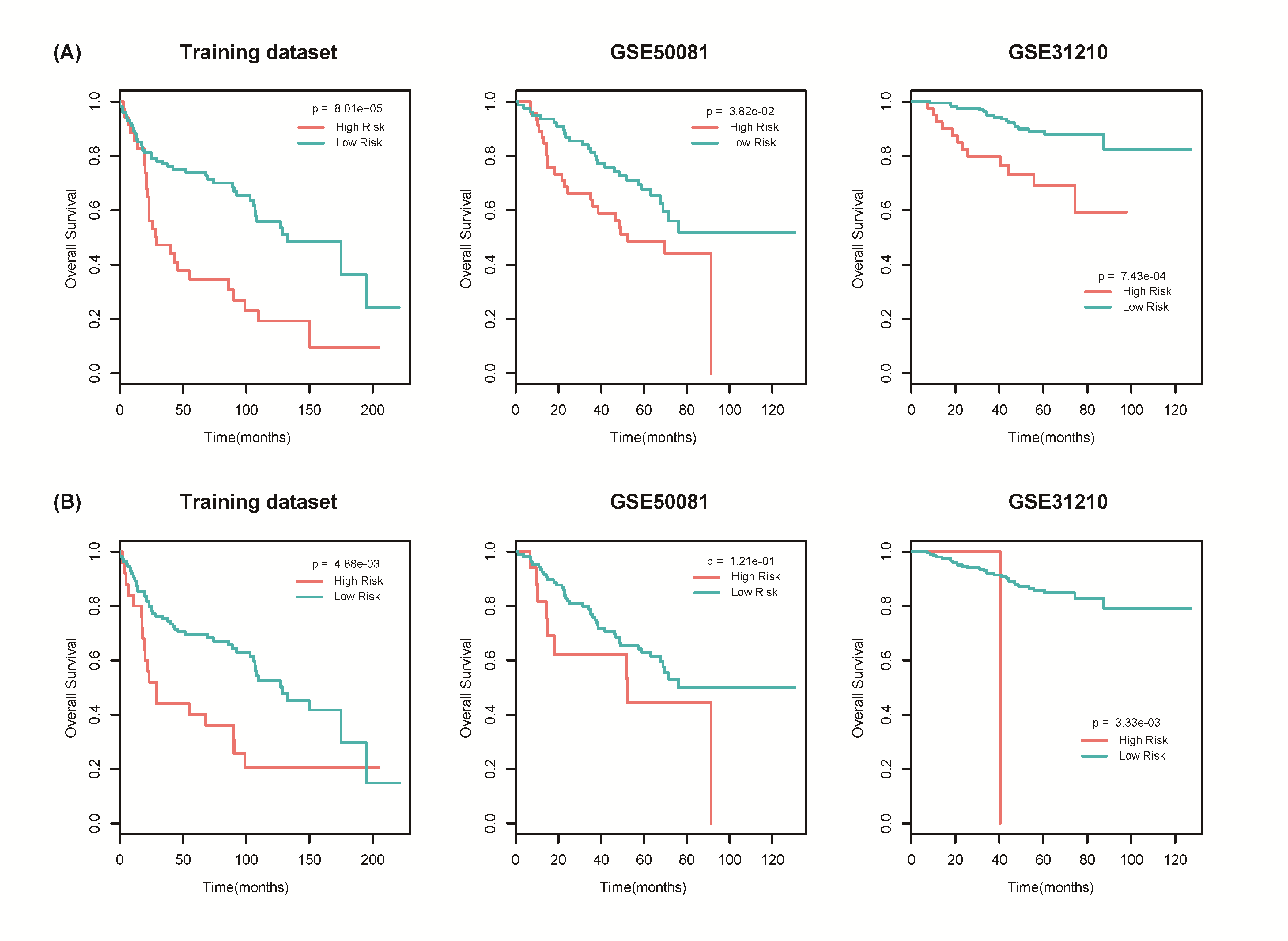


**Figure S3.** Kaplan-Meier estimates the overall survival in the training dataset and two independent validation datasets based on the differential expression of (A) *C1orf132* and (B) *TMPO-AS1*, respectively.


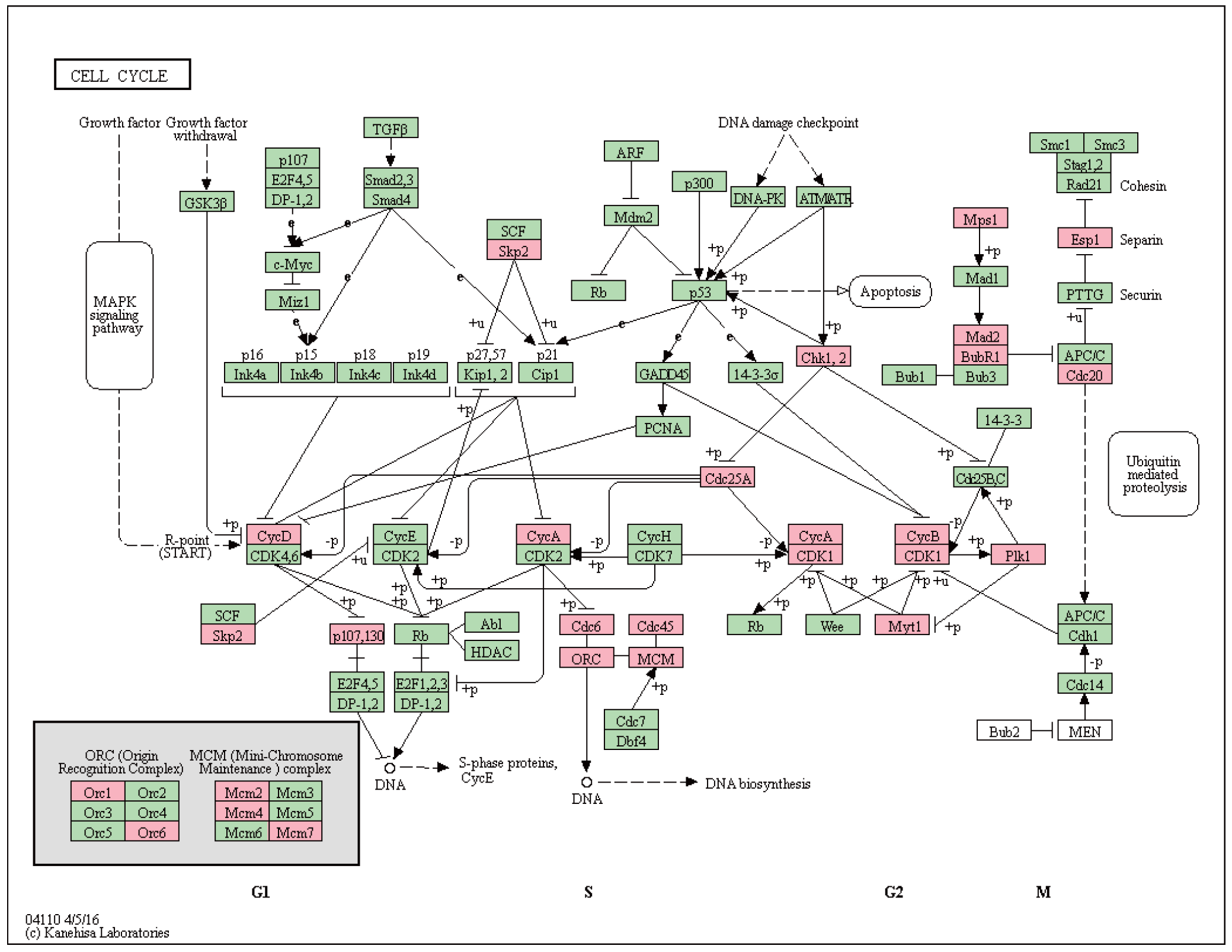


**Figure S4.** Cell cycle pathway annotated with differentially expressed genes in the KEGG database. Nodes with pink color are the genes that are differentially expressed in the high-risk patients compared with the low-risk patients separated by the lncRNA signature (*TMPO-AS1* and *C1orf132*).


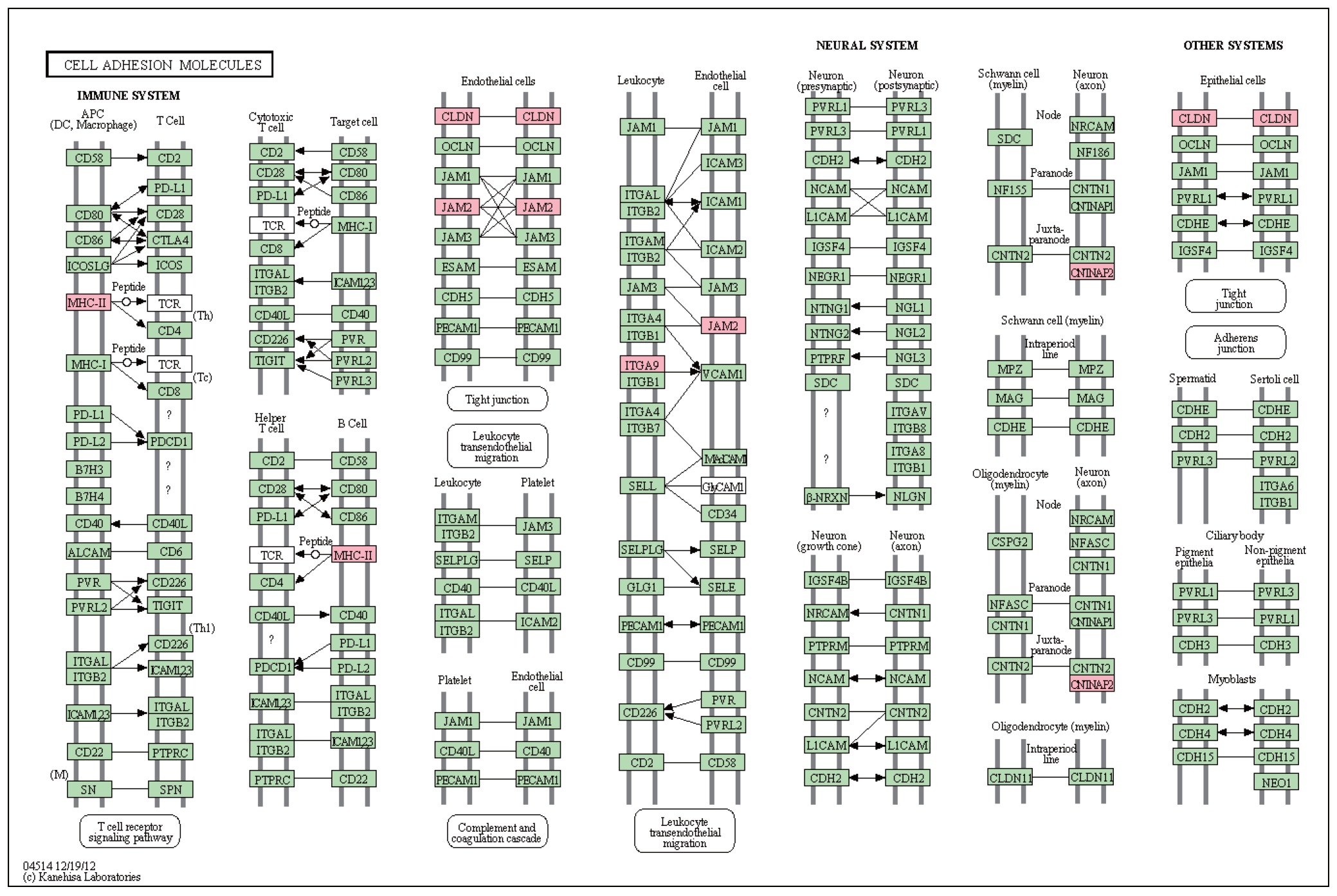


**Figure S5.** Cell adhesion molecules pathway annotated with differentially expressed genes in the KEGG database. Nodes with pink color are the genes that are differentially expressed in the high-risk patients compared with the low risk patients separated by the lncRNA signature (*TMPO-AS1* and *C1orf132*).


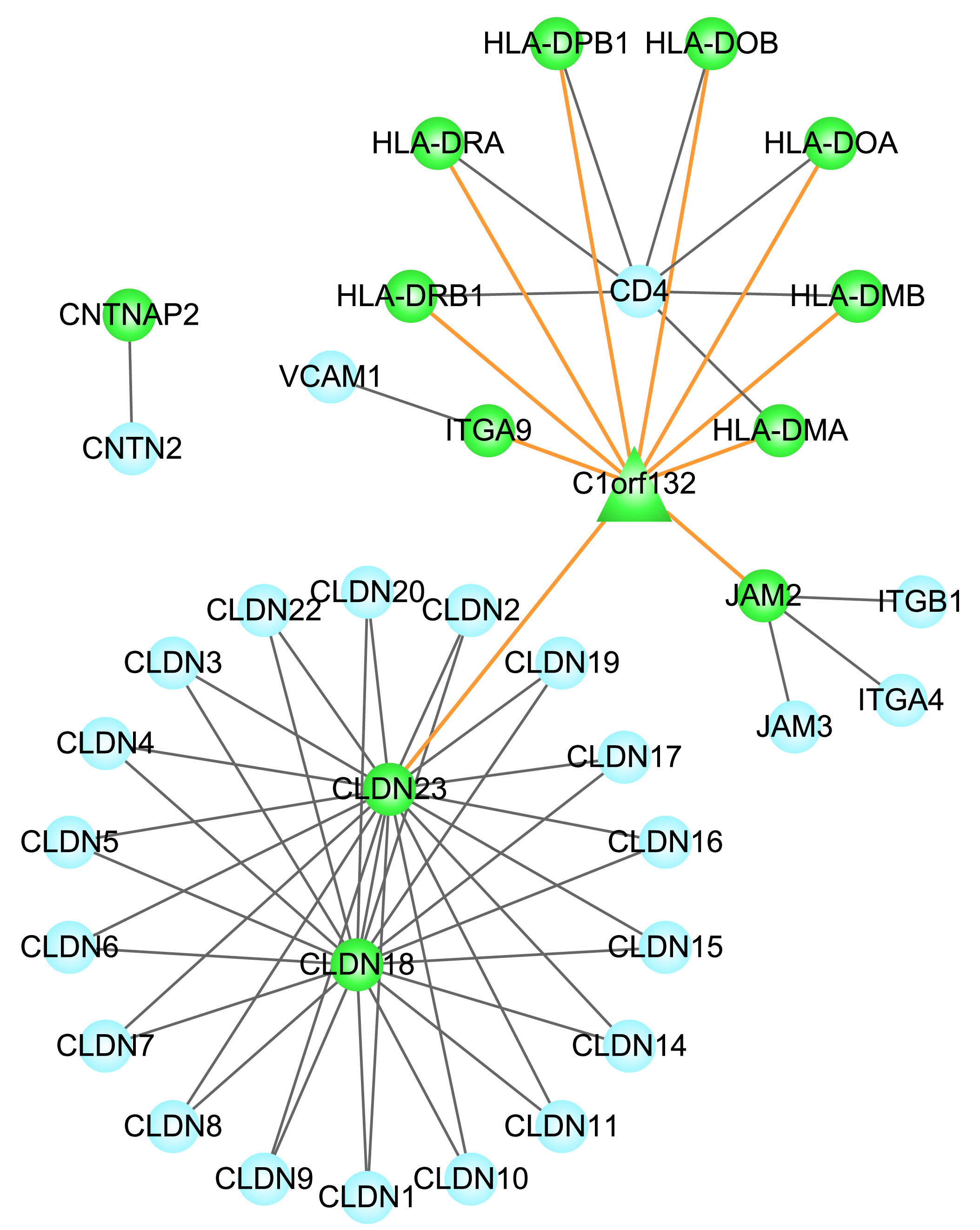


**Figure S6.** Sub-network of cell adhesion molecules pathway regulated by *C1orf132*. The triangles represent lncRNAs. The circles represent the cell adhesion molecules pathway genes. Nodes with green color represent that the genes or lncRNAs were down-regulated in high-risk cancer patients compared with the low-risk patients. The gray circles represent the genes that directly interact with differentially expressed genes in the cell adhesion molecules pathway, which are marked by gray lines. The orange lines represent the significantly co-expressed relationships between lncRNAs and differentially expressed genes.

**
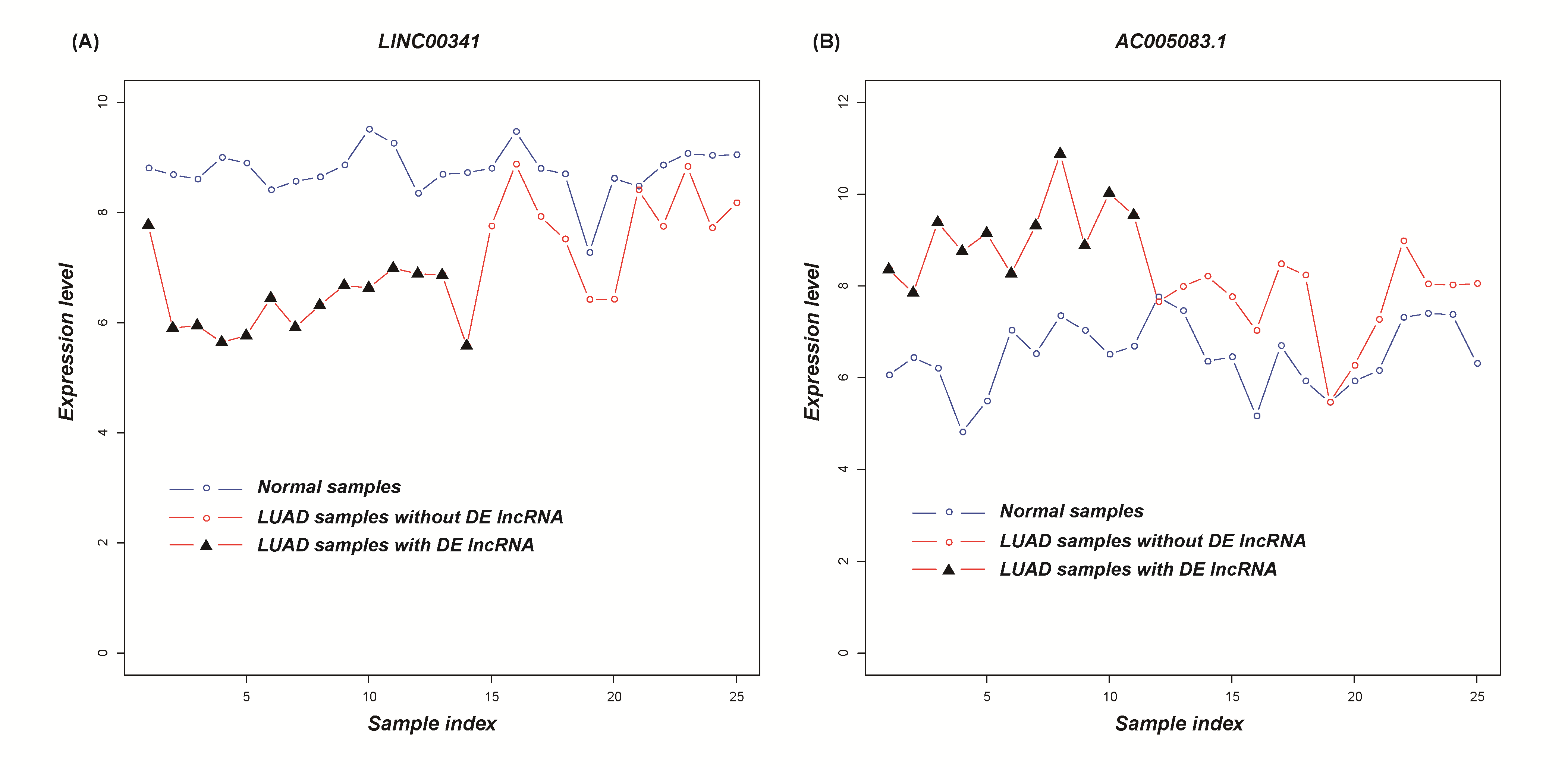
**

**Figure S7.** Expression levels of lncRNAs in the pair-wise LUAD patients for (A) *LINC00341* and (B) *AC005083.1*. X-axis is the number of cancer-normal sample pair. Y axis is the expression value of lncRNAs. Blue and red circles represent the normal samples and LUAD samples, respectively. The black triangles depict LUAD samples with differentially expressed lncRNA identified by *LincRIndiv*.
