# Supplementary material for: Differential expression analysis at the individual level reveals a lncRNA prognostic signature for lung adenocarcinoma
Source: Mol Cancer. 2017 Jun 6;16:98. doi: 10.1186/s12943-017-0666-z (PMC5461634; doi:10.1186/s12943-017-0666-z)
Supplement: Supplementary file 6 — Table S7. The consistency score under top 3, 5 and 7 reversal pairs in pair-wise datasets. Table S8. Comparison of LncRIndiv and RankComp methods using simulation data. Table S9. Information of lncRNAs with significant consistence between differential expression status and copy number alteration. Table S10. Information of genes co-expressed with TMPO-AS1 and C1orf132 in cell cycle pathway in GSE50081. Table S11. Differentially expressed lncRNAs identified by LncRIndiv method supported by experimental evidence. Table S12. Information of competing endogenous RNA and miRNA with the lncRNA C1orf132. Table S13.. Sensitivity, specificity, and F-score in simulated data under different scenarios. (DOC 257 kb) [file 12943_2017_666_MOESM6_ESM.doc]

**Table S7.** The consistency score under top 3, 5 and 7 reversal pairs in pair-wise datasets.

| **Dataset** | **3 pairs** | **5 pairs** | **7 pairs** |
| --- | --- | --- | --- |
| GSE27262 | 0.9673 | 0.9696 | 0.9664 |
| TCGA dataset | 0.9371 | 0.9399 | 0.9405 |

**Table S8. Comparison of *LncRIndiv* and *RankComp* methods using simulation data.**

| **|Log2FC^*^|** |  | **LncRIndiv** | **RankComp** |
| --- | --- | --- | --- |
| 1.0 | F-score | 1.0000 | 0.9496 |
|  | sensitivity | 1.0000 | 0.9080 |
|  | specificity | 1.0000 | 0.9953 |
| 1.5 | F-score | 0.9842 | 0.9519 |
|  | sensitivity | 0.9694 | 0.9102 |
|  | specificity | 0.9995 | 0.9975 |
| 2.0 | F-score | 0.9916 | 0.9562 |
|  | sensitivity | 0.9839 | 0.9200 |
|  | specificity | 0.9995 | 0.9953 |

^*^FC denotes fold change.

**Table S9.** Information of lncRNAs with significant consistence between differential expression status and copy number alteration.

| Ensembl_ID | Symbol | up/down | Copy number | | Reference |
| --- | --- | --- | --- | --- | --- |
| ENSG00000177406.4 | RP11-218M22.1 | down | loss |  | |
| ENSG00000177738.3 | CTD-2201E18.3 | up | gain |  | |
| ENSG00000179523.4 | EIF3J-AS1 | down | loss |  | |
| ENSG00000182873.4 | RP11-181G12.2 | down | loss |  | |
| ENSG00000188206.5 | HNRNPU-AS1 | up | gain |  | |
| ENSG00000196421.3 | LINC00176 | up | gain |  | |
| ENSG00000215068.3 | AC025171.1 | up | gain |  | |
| ENSG00000223393.1 | RP5-858B6.3 | up | gain |  | |
| ENSG00000224032.2 | EPB41L4A-AS1 | down | loss |  | |
| ENSG00000224660.1 | SH3BP5-AS1 | down | loss |  | |
| ENSG00000226920.1 | RP5-1068B5.3 | up | gain |  | |
| ENSG00000227502.2 | RP1-249H1.4 | down | loss |  | |
| ENSG00000227533.1 | SLC2A1-AS1 | up | gain |  | |
| ENSG00000228794.4 | RP11-206L10.11 | down | loss |  | |
| ENSG00000228889.2 | UBAC2-AS1 | up | gain |  | |
| ENSG00000229043.2 | AC091729.9 | up | gain |  | |
| ENSG00000229152.1 | ANKRD10-IT1 | up | gain |  | |
| ENSG00000229645.4 | LINC00341 | down | loss |  | |
| ENSG00000230487.3 | PSMG3-AS1 | up | gain |  | |
| ENSG00000230844.2 | ZNF674-AS1 | up | gain |  | |
| ENSG00000232977.2 | LINC00327 | down | loss |  | |
| ENSG00000233237.2 | LINC00472 | down | loss |  | |
| ENSG00000233461.1 | RP11-295G20.2 | up | gain |  | |
| ENSG00000233834.2 | AC005083.1 | up | gain |  | |
| ENSG00000234286.1 | AC006026.13 | up | gain |  | |
| ENSG00000234917.1 | RP5-994D16.3 | up | gain |  | |
| ENSG00000235280.2 | MCF2L-AS1 | up | gain |  | |
| ENSG00000235560.3 | AC002310.12 | up | gain |  | |
| ENSG00000235770.1 | LINC00607 | down | loss |  | |
| ENSG00000238005.2 | RP11-443B7.1 | up | gain |  | |
| ENSG00000240859.1 | AC093627.10 | up | gain |  | |
| ENSG00000241158.2 | ADAMTS9-AS1 | down | loss |  | |
| ENSG00000241684.1 | ADAMTS9-AS2 | down | loss |  | |
| ENSG00000245556.2 | CTD-2037K23.2 | down | loss |  | |
| ENSG00000245711.2 | NADK2-AS1 | up | gain |  | |
| ENSG00000247400.3 | DNAJC3-AS1 | up | gain |  | |
| ENSG00000247498.5 | RP11-392P7.6 | down | loss |  | |
| ENSG00000247556.2 | OIP5-AS1 | down | loss |  | |
| ENSG00000248092.3 | NNT-AS1 | up | gain |  | |
| ENSG00000248175.1 | CTC-428G20.3 | down | loss |  | |
| ENSG00000249673.2 | NOP14-AS1 | down | loss |  | |
| ENSG00000249700.4 | SRD5A3-AS1 | up | gain |  | |
| ENSG00000250802.2 | ZBED3-AS1 | down | loss |  | |
| ENSG00000253982.1 | CTD-2336O2.1 | down | loss |  | |
| ENSG00000254703.2 | FLI1-AS1 | down | loss |  | |
| ENSG00000255455.2 | RP11-890B15.3 | down | loss |  | |
| ENSG00000258441.1 | LINC00641 | down | loss |  | |
| ENSG00000258959.1 | RP11-1017G21.4 | down | loss |  | |
| ENSG00000260219.1 | RP11-347C12.10 | up | gain |  | |
| ENSG00000260279.1 | AC137932.5 | down | loss |  | |
| ENSG00000260920.1 | RP1-228H13.5 | up | gain |  | |
| ENSG00000261269.1 | RP11-389C8.2 | down | loss |  | |
| ENSG00000261786.1 | RP4-555D20.2 | down | loss |  | |
| ENSG00000261798.1 | RP1-118J21.25 | up | gain |  | |
| ENSG00000261799.1 | RP11-283I3.6 | down | loss |  | |
| ENSG00000264247.1 | LINC00909 | down | loss |  | |
| ENSG00000268001.1 | CTC-241F20.3 | down | loss |  | |
| ENSG00000268061.1 | NAPA-AS1 | down | loss |  | |
| ENSG00000269940.1 | RP11-73M18.7 | down | loss |  | |
| ENSG00000273015.1 | LINC00938 | down | loss |  | |
| ENSG00000273356.1 | RP11-804H8.6 | down | loss |  | |

**Table S10.** Information of genes co-expressed with *TMPO-AS1* and *C1orf132* in cell cycle pathway in GSE50081.

| lncRNA | Gene_Symbol | Gene ID | Direction^#^ | T-test p_value | Pearson r | Pearson p_value | Reference^*^ |
| --- | --- | --- | --- | --- | --- | --- | --- |
| C1orf132 | CCND3 | 896 | down | 1.33E-06 | 0.61 | 4.97E-06 |  |
| C1orf132 | RBL2 | 5934 | down | 3.58E-03 | 0.73 | 3.04E-09 |  |
| TMPO-AS1 | CDC20 | 991 | up | 7.46E-06 | 0.48 | 4.14E-02 |  |
| TMPO-AS1 | CDC25A | 993 | up | 1.58E-05 | 0.51 | 3.25E-02 |  |
| TMPO-AS1 | ESPL1 | 9700 | up | 5.65E-04 | 0.61 | 7.32E-03 |  |
| TMPO-AS1 | MCM4 | 4173 | up | 1.95E-03 | 0.61 | 6.98E-03 |  |
| TMPO-AS1 | MCM7 | 4176 | up | 2.41E-03 | 0.58 | 1.22E-02 |  |
| TMPO-AS1 | MCM2 | 4171 | up | 2.75E-03 | 0.57 | 1.29E-02 |  |

^#^The direction of genes mean up-regulation or down-regulation in the high-risk patient group compared with the low-risk patient group.

^*^The references reported the roles of genes related with cancer progression. The correlation of *CCND3*, *RBL2*, *CDC20* and *CDC25A* with lung cancer have been described in the main text. The up-regulation of *ESPL1* , *MCM2* , *MCM7* were significantly associated with poor prognosis for patients with non-small cell lung cancer . Biological experiment showed that down-regulation of *MCM4* reduced proliferation of non-small cell lung cancer cells .

**Table S11.** Differentially expressed lncRNAs of LUAD identified by *LncRIndiv* method supported by experimental evidence.

| Ensemble ID | Symbol | up/down | Reference |
| --- | --- | --- | --- |
| ENSG00000180139.10 | ACTA2-AS1 | up |  |
| ENSG00000248771.1 | RP11-294O2.2 | up |  |
| ENSG00000242808.3 | SOX2-OT | up |  |
| ENSG00000224259.1 | RP11-48O20.4 | up |  |
| ENSG00000260940.1 | RP4-575N6.5 | down |  |
| ENSG00000261496.1 | RP13-514E23.1 | down |  |
| ENSG00000130600.11 | H19 | down |  |

**Table S12. Information of competing endogenous RNA and miRNA with the lncRNA *C1orf132*.**

| LncRNA | miRNA | Gene | miRNA-lncRNA  source database | miRNA-gene  source database |
| --- | --- | --- | --- | --- |
| C1orf132 | hsa-miR-33a | CCND3 | mircode | miranda |
| C1orf132 | hsa-miR-16 | CCND3 | mircode | miranda;targetscan |
| C1orf132 | hsa-miR-370 | CCND3 | miranda | miranda |
| C1orf132 | hsa-miR-24 | CCND3 | mircode | miranda |
| C1orf132 | hsa-miR-7 | CCND3 | mircode | miranda |
| C1orf132 | hsa-miR-384 | CCND3 | miranda | miranda |
| C1orf132 | hsa-miR-1907 | CCND3 | mircode | targetscan |
| C1orf132 | hsa-miR-15a | CCND3 | mircode | miranda |
| C1orf132 | hsa-miR-506 | CCND3 | mircode | miranda;targetscan |
| C1orf132 | hsa-miR-124 | CCND3 | mircode | miranda;targetscan |
| C1orf132 | hsa-miR-497 | CCND3 | mircode | miranda;targetscan |
| C1orf132 | hsa-miR-15b | CCND3 | mircode | miranda |
| C1orf132 | hsa-miR-138 | CCND3 | mircode | miranda;targetscan |
| C1orf132 | hsa-miR-424 | CCND3 | mircode | miranda;targetscan |
| C1orf132 | hsa-miR-195 | CCND3 | mircode | miranda;targetscan |
| C1orf132 | hsa-miR-302e | RBL2 | mircode | miranda |
| C1orf132 | hsa-miR-106a | RBL2 | mircode | miranda |
| C1orf132 | hsa-miR-200a | RBL2 | mircode | miranda |
| C1orf132 | hsa-miR-340 | RBL2 | miranda | miranda;targetscan |
| C1orf132 | hsa-miR-17-5p | RBL2 | mircode | mirtarbase;targetscan |
| C1orf132 | hsa-miR-20b | RBL2 | mircode | miranda |
| C1orf132 | hsa-miR-370 | RBL2 | miranda | miranda |
| C1orf132 | hsa-miR-373 | RBL2 | mircode | miranda |
| C1orf132 | hsa-miR-302a | RBL2 | mircode | miranda |
| C1orf132 | hsa-miR-129-5p | RBL2 | mircode | miranda |
| C1orf132 | hsa-miR-302b | RBL2 | mircode | miranda |
| C1orf132 | hsa-miR-141 | RBL2 | mircode | miranda |
| C1orf132 | hsa-miR-20a | RBL2 | mircode | miranda |
| C1orf132 | hsa-miR-106b | RBL2 | mircode | miranda |
| C1orf132 | hsa-miR-107 | RBL2 | mircode | miranda |
| C1orf132 | hsa-miR-204 | RBL2 | mircode | miranda |
| C1orf132 | hsa-miR-211 | RBL2 | mircode | miranda |
| C1orf132 | hsa-miR-93 | RBL2 | mircode | miranda;targetscan |
| C1orf132 | hsa-miR-519d | RBL2 | mircode | miranda;targetscan |
| C1orf132 | hsa-miR-17 | RBL2 | mircode | miranda |
| C1orf132 | hsa-miR-372 | RBL2 | mircode | miranda |
| C1orf132 | hsa-miR-520e | RBL2 | mircode | miranda |
| C1orf132 | hsa-miR-302c | RBL2 | mircode | miranda |
| C1orf132 | hsa-miR-520b | RBL2 | mircode | miranda |
| C1orf132 | hsa-miR-302d | RBL2 | mircode | miranda |

**Table S13.** Sensitivity, specificity, and F-score in simulated data under different scenarios.

| **Sample Number** | | **210 vs 210** | | | **60 vs 60** | | |
| --- | --- | --- | --- | --- | --- | --- | --- |
| |Log2FC^*^| | Grade^#^ | F-score | Sensitivity | Specificity | F-score | Sensitivity | Specificity |
| 1.0 | 10 | 1.0000 | 1.0000 | 1.0000 | 0.9800 | 0.9639 | 0.9967 |
|  | 20 | 0.8527 | 0.7445 | 0.9978 | 0.8239 | 0.7019 | 0.9973 |
|  | 30 | 0.8368 | 0.7206 | 0.9978 | 0.7673 | 0.6229 | 0.9987 |
| 1.5 | 10 | 0.9842 | 0.9694 | 0.9995 | 0.976 | 0.9548 | 0.9982 |
|  | 20 | 0.9209 | 0.8552 | 0.9975 | 0.9102 | 0.8365 | 0.9982 |
|  | 30 | 0.8931 | 0.8081 | 0.998 | 0.8696 | 0.7699 | 0.999 |
| 2.0 | 10 | 0.9916 | 0.9839 | 0.9995 | 0.9872 | 0.9748 | 1.0000 |
|  | 20 | 0.9611 | 0.9272 | 0.9975 | 0.9629 | 0.9296 | 0.9987 |
|  | 30 | 0.9560 | 0.9173 | 0.9980 | 0.9520 | 0.9093 | 0.9990 |

^*^FC denotes fold change.

^#^denotes the number of disease samples in which a DE lncRNA was simulated to be differentially expressed.

**Reference**

1. Yabuta N, Onda H, Watanabe M, Yoshioka N, Nagamori I, Funatsu T et al. Isolation and characterization of the TIGA genes, whose transcripts are induced by growth arrest. Nucleic Acids Res. 2006;34(17):4878-92.

2. Shen Y, Katsaros D, Loo LW, Hernandez BY, Chong C, Canuto EM et al. Prognostic and predictive values of long non-coding RNA LINC00472 in breast cancer. Oncotarget. 2015;6(11):8579-92.

3. Joo JH, Ryu D, Peng Q, Sugrue SP. Role of Pnn in alternative splicing of a specific subset of lncRNAs of the corneal epithelium. Mol Vis. 2014;20:1629-42.

4. Yao J, Zhou B, Zhang J, Geng P, Liu K, Zhu Y et al. A new tumor suppressor LncRNA ADAMTS9-AS2 is regulated by DNMT1 and inhibits migration of glioma cells. Tumour Biol. 2014;35(8):7935-44.

5. Kim J, Abdelmohsen K, Yang X, De S, Grammatikakis I, Noh JH et al. LncRNA OIP5-AS1/cyrano sponges RNA-binding protein HuR. Nucleic Acids Res. 2016;44(5):2378-92.

6. Wang L, Li Z, Li Z, Yu B, Wang Y. Long noncoding RNAs expression signatures in chondrogenic differentiation of human bone marrow mesenchymal stem cells. Biochem Biophys Res Commun. 2015;456(1):459-64.

7. Han LP, Fu T, Lin Y, Miao JL, Jiang QF. MicroRNA-138 negatively regulates non-small cell lung cancer cells through the interaction with cyclin D3. Tumour Biol. 2016;37(1):291-8.

8. Schaffer BE, Park KS, Yiu G, Conklin JF, Lin C, Burkhart DL et al. Loss of p130 accelerates tumor development in a mouse model for human small-cell lung carcinoma. Cancer Res. 2010;70(10):3877-83.

9. Kato T, Daigo Y, Aragaki M, Ishikawa K, Sato M, Kaji M. Overexpression of CDC20 predicts poor prognosis in primary non-small cell lung cancer patients. J Surg Oncol. 2012;106(4):423-30.

10. Lin TC, Lin PL, Cheng YW, Wu TC, Chou MC, Chen CY et al. MicroRNA-184 Deregulated by the MicroRNA-21 Promotes Tumor Malignancy and Poor Outcomes in Non-small Cell Lung Cancer via Targeting CDC25A and c-Myc. Ann Surg Oncol. 2015;22 Suppl 3:S1532-9.

11. Zhang C, Min L, Zhang L, Ma Y, Yang Y, Shou C. Combined analysis identifies six genes correlated with augmented malignancy from non-small cell to small cell lung cancer. Tumour Biol. 2016;37(2):2193-207.

12. Kikuchi J, Kinoshita I, Shimizu Y, Kikuchi E, Takeda K, Aburatani H et al. Minichromosome maintenance (MCM) protein 4 as a marker for proliferation and its clinical and clinicopathological significance in non-small cell lung cancer. Lung Cancer. 2011;72(2):229-37.

13. Toyokawa G, Masuda K, Daigo Y, Cho HS, Yoshimatsu M, Takawa M et al. Minichromosome Maintenance Protein 7 is a potential therapeutic target in human cancer and a novel prognostic marker of non-small cell lung cancer. Mol Cancer. 2011;10:65.

14. Yang J, Ramnath N, Moysich KB, Asch HL, Swede H, Alrawi SJ et al. Prognostic significance of MCM2, Ki-67 and gelsolin in non-small cell lung cancer. BMC Cancer. 2006;6:203.

15. Zhang L, Zhou XF, Pan GF, Zhao JP. Enhanced expression of long non-coding RNA ZXF1 promoted the invasion and metastasis in lung adenocarcinoma. Biomed Pharmacother. 2014;68(4):401-7.

16. Wang G, Chen H, Liu J. The long noncoding RNA LINC01207 promotes proliferation of lung adenocarcinoma. American journal of cancer research. 2015;5(10):3162-73.

17. Hou Z, Zhao W, Zhou J, Shen L, Zhan P, Xu C et al. A long noncoding RNA Sox2ot regulates lung cancer cell proliferation and is a prognostic indicator of poor survival. The international journal of biochemistry & cell biology. 2014;53:380-8.

18. Zhang J, Zhu N, Chen X. A novel long noncoding RNA LINC01133 is upregulated in lung squamous cell cancer and predicts survival. Tumour Biol. 2015;36(10):7465-71.

19. Xu G, Chen J, Pan Q, Huang K, Pan J, Zhang W et al. Long noncoding RNA expression profiles of lung adenocarcinoma ascertained by microarray analysis. PloS one. 2014;9(8):e104044.

20. Song H, Sun W, Ye G, Ding X, Liu Z, Zhang S et al. Long non-coding RNA expression profile in human gastric cancer and its clinical significances. J Transl Med. 2013;11:225.
